# Communication About End of Life for Patients Living With Amyotrophic Lateral Sclerosis: A Scoping Review of the Empirical Evidence

**DOI:** 10.3389/fneur.2021.683197

**Published:** 2021-08-04

**Authors:** Shelagh K. Genuis, Westerly Luth, Sandra Campbell, Tania Bubela, Wendy S. Johnston

**Affiliations:** ^1^Division of Neurology, Department of Medicine, University of Alberta, Edmonton, AB, Canada; ^2^School of Public Health, University of Alberta, Edmonton, AB, Canada; ^3^John W. Scott Health Sciences Library, Faculty of Medicine and Dentistry, University of Alberta, Edmonton, AB, Canada; ^4^Faculty of Health Sciences, Simon Fraser University, Burnaby, BC, Canada

**Keywords:** advance care planning, amyotrophic lateral sclerosis, health communication, palliative care, terminal care, review

## Abstract

**Background:** Communication about end of life, including advance care planning, life-sustaining therapies, palliative care, and end-of-life options, is critical for the clinical management of amyotrophic lateral sclerosis patients. The empirical evidence base for this communication has not been systematically examined.

**Objective:** To support evidence-based communication guidance by (1) analyzing the scope and nature of research on health communication about end of life for amyotrophic lateral sclerosis; and (2) summarizing resultant recommendations.

**Methods:** A scoping review of empirical literature was conducted following recommended practices. Fifteen health-related and three legal databases were searched; 296 articles were screened for inclusion/exclusion criteria; and quantitative data extraction and analysis was conducted on 211 articles with qualitative analysis on a subset of 110 articles that focused primarily on health communication. Analyses summarized article characteristics, themes, and recommendations.

**Results:** Analysis indicated a multidisciplinary but limited evidence base. Most reviewed articles addressed end-of-life communication as a peripheral focus of investigation. Generic communication skills are important; however, substantive and sufficient disease-related information, including symptom management and assistive devices, is critical to discussions about end of life. Few articles discussed communication about specific end-of-life options. Communication recommendations in analyzed articles draw attention to communication processes, style and content but lack the systematized guidance needed for clinical practice.

**Conclusions:** This review of primary research articles highlights the limited evidence-base and consequent need for systematic, empirical investigation to inform effective communication about end of life for those with amyotrophic lateral sclerosis. This will provide a foundation for actionable, evidence-based communication guidelines about end of life. Implications for research, policy, and practice are discussed.

## Introduction

Communication about advance care planning, life-sustaining therapies, palliative care and other options in the last months of life is central to the clinical management of fatal neurological diseases, such as amyotrophic lateral sclerosis (ALS) (1–5). ALS is a degenerative motor neuron disease characterized by progressive motor impairment leading to severe disability and eventual respiratory failure (6). ALS incidence is between 0.6 and 3.8 per 100,000 person-years and its prevalence is 4.1–8.4 per 100,000 persons (7); it is considered a “rare disease” (8). Patients living with ALS confront significant practical and existential losses (9–12) as they contend with an uncertain and variable disease trajectory, a median overall survival of 30 months after symptom onset, and a 5–10% survival rate one decade after diagnosis (13, 14). Accordingly, there is a need for clear and frequent communication with patients and their families over the course of the disease (15).

Timely and ongoing discussion about end of life (including advance care planning, technology for symptom management, palliative care, and other end of life options) is particularly important for ALS patients. Therapies introduced for symptom management, such as non-invasive ventilation, may rapidly become life-sustaining, thus changing the natural disease trajectory and making it difficult to predict when a patient is entering the last months of life (16, 17). Further, many patients experience substantial functional communication and cognitive difficulties, which may interfere with communication at later stages of the disease (6). Effective discussions about end of life help alleviate anticipatory fears, especially around choking (6); guide decisions about life-sustaining therapies (18–20); facilitate decisions that are consistent with patients' and families' priorities and needs over time (6, 21, 22); and preserve patient autonomy and dignity (23).

Compounding a complex communication environment and in the ongoing absence of a cure or treatment, ALS is perceived by patients and their families as a “death sentence” (24), “the self under attack” or a “downward journey” (25). This is in contrast to the empowering representation of “fighting” diseases with multiple treatment options, such as many cancers (26, 27). Moreover, increasing discussion and legalization of voluntary assisted death across jurisdictions, including both physician-assisted suicide and euthanasia (28–30), and a focus on ALS in court cases, case studies published in medical journals, and media portrayals of voluntary assisted death (31–35) raises the possibility that this option may become the focus of end-of-life discussions with ALS patients, highlighting the need for effective communication about end-of-life decision-making.

Consensus-based guidelines from Canada, Europe and the United States recommend discussing preferences for life-sustaining therapies and end-of-life care on a regular basis with ALS patients (16, 36, 37). However, guidelines for discussions about end of life with ALS patients have not been published. Communication guidelines have focused on the disclosure of the ALS diagnosis, offering clinicians specific guidance for introducing and discussing the challenges of this rapidly progressing, neurodegenerative disease (36, 38, 39).

Published reviews focusing on quality of care and quality of life (40), end-of-life management (41), and palliative care information needs of ALS patients (42) have also drawn attention to the importance of communication about end of life for people living with ALS. However, there is need for a structured, systematic, and evidence-informed approach to this communication (43). Given the recognition that research evidence is as important in palliative care as it is in other fields of medicine (44), this scoping review investigates the scope and nature of empirical articles on communication about end of life with ALS patients, identifies gaps, and provides a foundation for empirically-based, communication guidelines for discussions about end of life with ALS patients.

## Methods

### Identification of Research Question

A team of experts from fields including neurology and health communication were consulted to identify goals and research questions for this scoping review. Identified goals were to understand the empirical evidence base, identify research gaps, determine research opportunities, and provide a foundation for clinically focused communication guidelines. Specifically, the review addressed two research questions: (1) What is the scope and nature of published research on ALS and health communication about end of life? And (2) what, if any, recommendations are made in primary research articles whose central focus is end-of-life communication with patients living with ALS?

### Design

Scoping reviews are commonly undertaken when there is a broad question, a range of study designs, no prior knowledge synthesis on the topic, and an interest in identifying gaps and envisaging future research directions (45–48). The methodology used for this review was based on Arksey and O'Malley's five stages for scoping reviews: (i) identify the research question; (ii) develop the search strategy; (iii) apply inclusion and exclusion criteria to select articles; (iv) chart and collate the data; and (v) summarize and report the results (45). In accordance with recommendations for scoping reviews (47, 48), a quantitative, numerical summary analysis, followed by a qualitative thematic analysis of the subset of articles whose central focus was communication in the context of ALS and end of life was conducted. The discussion section completes the summary and reporting stage as it focuses on the meaning and implications of the study findings (47).

### Data Sources and Search Strategy

An expert health sciences librarian developed search strategies for the following electronic databases: MEDLINE (Ovid), EMBASE (Ovid), PsycINFO, CINAHL (EBSCO), SCOPUS, Dissertations and Theses Global (Proquest), and Web of Science, and EMB Reviews (Ovid) including Cochrane Database of Systematic Reviews, ACP Journal Club, Database of Abstracts of Reviews of Effects, Cochrane Central Register of Controlled Trials, Cochrane Methodology Register, Health Technology Assessment, and NHS Economic Evaluation Database. The following legal databases were also searched: Westlaw, Heinonline and the Factiva subcategory “US law reviews and journals.” Search algorithms used controlled vocabulary within databases and synonyms for “amyotrophic lateral sclerosis,” “end of life,” and “health communication.” Date or other limits were not applied. Initial searches were completed in October 2015 and updated in January 2018. A second update was conducted in May 2021. At the time of the second update, all the EMB Reviews (Ovid) databases had been replaced by Cochrane Library (CDSR and Central Register of Controlled Trials). The search strategy used for Medline is included as a sample in Supplementary File 1; other detailed search strategies are available from the corresponding author.

### Application of Inclusion/Exclusion Criteria

Article records, including titles and abstracts, were retrieved and uploaded to bibliographic management software (Endnote 7). For the initial search and 2018 update, four coders removed duplicates and applied the inclusion/exclusion criteria to the article records that met the search criteria. Articles meeting the following criteria were included: (1) reported primary quantitative and/or qualitative empirical data; (2) addressed end of life for people with ALS; (3) discussed health communication; and, (4) were published in the English language. Health communication was defined as per the Medical Subject Headings (MeSH) thesaurus: the transfer of information from experts in the medical and public health fields to patients and the public, and the study or use of communication strategies to inform and influence health-related decisions (49). All coders received training and discrepancies were resolved through discussion to consensus during the training period. Coders then screened 10% of the article records and inter-coder reliability was determined by calculating the Light's kappa coefficient in Microsoft Excel as 0.87. Each coder independently screened one quarter of the remaining records. This same process was followed when assessing the full text articles. Based on 10% of the articles the Light's Kappa coefficient was calculated as 0.81. Two coders completed the 2021 update. The Kappa coefficient was calculated as 0.99 for screening the article records and coding the included full text articles.

### Quantitative Data Extraction and Analysis

Based on the research questions and expert input a web-based, standardized data extraction sheet was developed. Each selected article was coded for: bibliographic information, jurisdiction where the study was conducted, research design, study methods, participant population, sample size, quality of life and family burden, discussion of voluntary assisted death, and peripheral or primary focus on health communication. Three trained coders extracted data from the selected full text articles. The calculated Light's Kappa coefficient was 0.74. The Kappa coefficient for the 2021 update was 0.99. Numerical summary analysis was conducted based on the data extracted to a priori categories (47, 50).

### Qualitative Analysis and Synthesis

Qualitative, inductive analysis was conducted on the subset of articles that were coded during quantitative analysis as having primary focus on health communication (the “communication subset”). Based on the research questions and expert input, key concepts and themes were identified using an iterative approach. Discussion to consensus was achieved by working through a small sample of articles. One coder coded the communication subset; the second coder coded 10% of the articles. Based on this 10%, the Kappa coefficient was assessed as 0.97 (initial and 2018 update) and 0.94 (2021 update) for the qualitative analysis. NVivo 10 software facilitated data organization and qualitative coding.

## Results

### Study Screening and Inclusion

**Figure 1:**
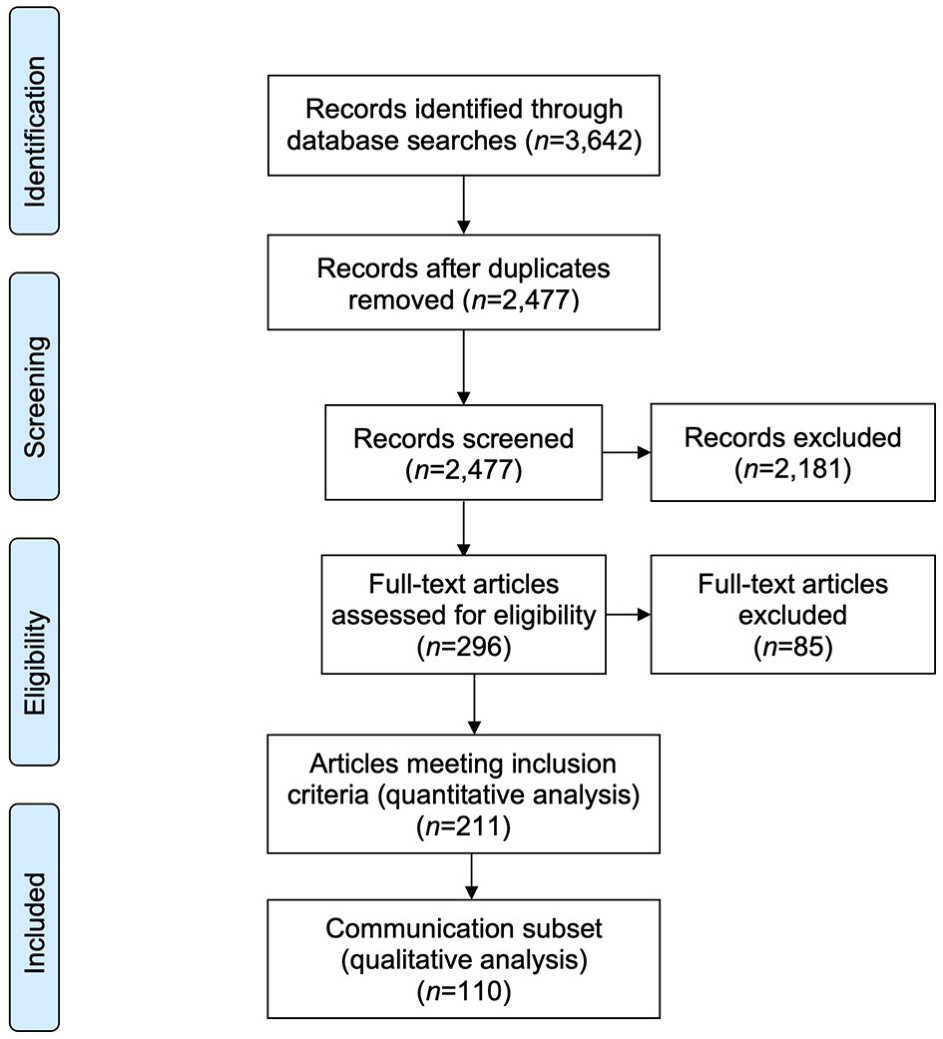
PRISMA flow: Article selection process.

Literature searches returned 2,477 unique article records, of which 296 were potentially relevant and eligible for full-text review. Of these, 211 met the review's inclusion criteria for quantitative analysis. (See Supplementary File 2 for list of included studies). One hundred and ten articles focused explicitly on health communication. These comprised the ‘communication subset’ and were included in qualitative thematic analysis (Figure 1).

### Quantitative Analysis of all Articles (*n* = 211)

#### Distribution of Articles

**Figure 2:**
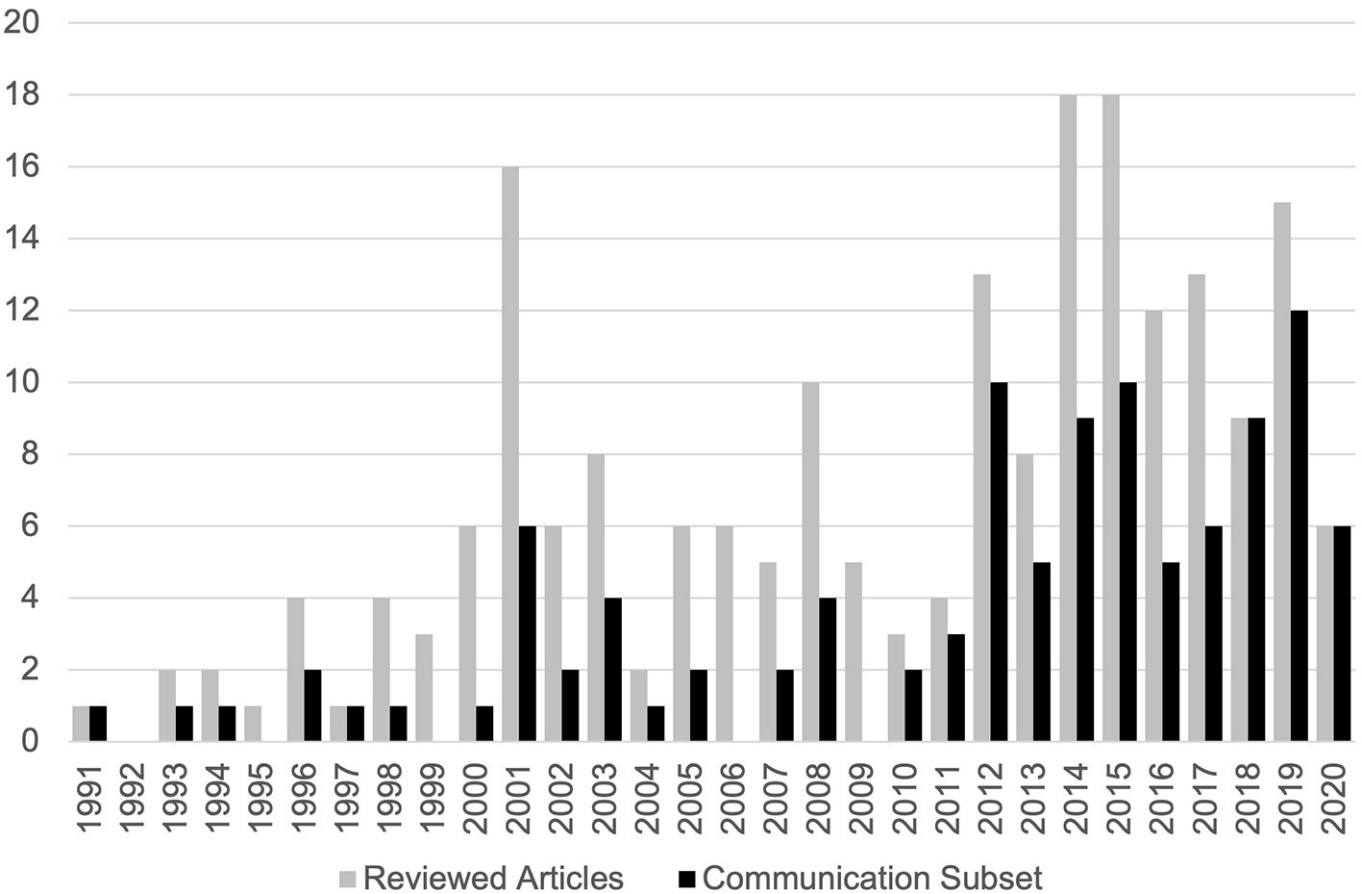
Distribution of reviewed articles and communication subset by year.

There was a modest upward trend in publications from 1991 to 2020 (Figure 2), with the majority of publications published after 2011 (51%) and peaks in 2014 and 2015. Four articles from the first 4 months of 2021 met the inclusion criteria. The reviewed articles (*n* = 211) were published in 84 different journals. Articles in the communication subset (*n* = 110) were published in 60 different journals. Seven journals published more than five reviewed articles each and almost 50% (*n* = 105) of the reviewed articles (Table 1). Reviewed articles were primarily published in journals identified by five non-exclusive Web of Science journal subject categories (Table 2). Eleven articles were published in journals not indexed by Web of Science.

**Table 1:** Distribution by journal title.

**Journal title**	**Reviewed**	**Communication focus**
Neurology	28	4
Amyotrophic Lateral Sclerosis and Frontotemporal Degeneration^*^	28	13
Journal of the Neurological Sciences	17	9
Palliative Medicine	10	8
Journal of Neurology	8	3
Journal of Pain and Symptom Management	7	3
Palliative and Supportive Care	7	4

* *formerly indexed as Amyotrophic Lateral Sclerosis and Other Motor Neuron Disorders (2000–2004) and Amyotrophic Lateral Sclerosis (2005–2012)*.

**Table 2:** Distribution by Web of Science journal subject category.

**Journal subject category**	**Reviewed articles (*n* = 211)**
Clinical Neurology-SCIE^*^	105
Health Care Sciences & Services-SCIE	40
Medicine, General & Internal-SCIE	19
Public Environmental & Occupational Health-SSCI^**^	19
Health Policy & Services-SSCI	10
Other (*n* = 25 subject categories)	40

* *Science Citation Index*.

** *Social Science Citation Index*.

#### Article Characteristics

Table 3 summarizes the general characteristics of the included articles (*n* = 211). In addition to the United States, European Union, United Kingdom, and Canada, studies were conducted in Japan, Taiwan, Singapore, Korean, Australia, and Israel. Studies used quantitative, qualitative, and mixed methods. Most included ALS patients or family members, with both groups being included in 69 articles. Study sample sizes ranged from two (a qualitative document analysis) to 1,636 (administrative data analysis). The 42 articles with healthcare professionals as participants included small interview-based studies (<35 participants), larger questionnaire-based studies (>100 participants), and studies that focused on care teams in multidisciplinary clinics.

**Table 3:** Study characteristics.

**Study characteristic**	***n* = 211 (%)**
Jurisdiction^*^	
United States	73 (34.6%)
European Union	71 (33.6%)
United Kingdom	23 (10.9%)
Canada	16 (7.6%)
Other	37 (17.5%)
**Study design**	
Quantitative	132 (62.6%)
Qualitative	55 (26.1%)
Mixed methods	24 (11.4%)
Study methods^*^	
Quantitative methods	
Questionnaire	112 (53.1%)
Cohort study	39 (18.5%)
Other quantitative methods^**^	25 (11.8%)
Clinical trial	7 (3.3%)
Case control	7 (3.3%)
Qualitative methods	
Interview	61 (28.9%)
Other qualitative methods^**^	9 (4.3%)
Document analysis	6 (2.8%)
Focus group	6 (2.8%)
Case study	2 (0.9%)
Participants^*^	
ALS patients	160 (75.8%)
Family members/informal caregivers	94 (44.5%)
Health care professionals	42 (19.9%)
General population	3 (1.4%)

* *Articles from multiple jurisdictions or using multiple methods are included in each relevant category*.

** *Articles using quantitative or qualitative methods not included in the data extraction sheet, for example, health economic analysis and chart review*.

#### Quality of Life

Of the 211 articles, 68% (*n* = 144) addressed quality of life (QoL) as experienced by patients (*n* = 120), or the perspectives of family (*n* = 41) and healthcare professionals (*n* = 11) on patients' QoL. These articles addressed the physical domain (*n* = 116), psychological/emotional domain (*n* = 104), social functioning domain (*n* = 60), religious/spiritual domain (*n* = 41), and financial domain (*n* = 31); 107 articles discussed more than one domain.

#### Family Burden

The articles (*n* = 82) that addressed family burden addressed burden associated with the psychological/emotional domain (*n* = 69), social functioning domain (*n* = 31), physical domain (*n* = 30), financial domain (*n* = 22), and unspecified domain (*n* = 28); 58 articles addressed more than one domain. Three articles addressed the psychological/emotional burden associated with concerns about familial ALS genetic risk. Sixty-five articles addressed both QoL for patients living with ALS and family burden, and 20 articles identified changes in family dynamics as a factor in patient QoL and/or family burden.

#### End of Life and Voluntary Assisted Dying

One hundred and twenty-three articles included the views or perspectives of ALS patients, family members, and/or healthcare professionals about end-of-life choices or options (Table 4), including palliative care, withdrawal of treatment, palliative sedation, and voluntary assisted death. The majority of articles focused on end-of-life options without discussing voluntary assisted death (60.6%; *n* = 77); 6.5% (*n* = 8) articles focused on voluntary assisted death exclusively.

**Table 4:** Views represented in articles discussing end-of-life options and/or voluntary assisted death.

***n* = 123 (%)**	**ALS patients**	**Family members**	**Health professionals**	**End-of-life options/voluntary assisted death**
123	101	39	30	End-of-life options discussed
77	61	29	20	End-of-life options; not voluntary assisted death
38	33	7	9	End-of-life options, including voluntary assisted death
8	7	3	1	Voluntary assisted death; not other end-of-life options

### Quantitative and Qualitative Analysis of the Health Communication Subset (*n* = 110)

Eighty-one articles within the health communication subset (*n* = 110) highlighted the importance of discussions about end of life for people living with ALS. Twenty-eight articles noted the influence of communication on patient care, and 19 noted its influence on the therapeutic relationship between healthcare professionals and patients and/or their families. Fifty-five articles discussed communication about specific end-of-life options; 13 noted voluntary assisted death. Thirty-three articles included discussion of symptom management at end of life, for example, nutritional or respiratory support. Themes identified in the communication subset included communication quality (communication barriers and facilitators) (*n* = 81), difficult conversations (*n* = 72), and functional communication challenges (*n* = 45) (Table 5).

**Table 5:** Themes identified in the health communication subset.

**Themes**	***N* = 110 (%)**	**References**
**Communication barriers**	57 (51.8%)	(18, 19, 39, 51–104)
Insufficient information given about disease and/or assistive devices	31 (28.2%)	(18, 52, 54–58, 60–63, 65–67, 69, 74, 75, 79, 81, 83–85, 87, 89, 90, 92, 96–98, 102, 105)
Lack of options communicated by Health professionals (symptom management and/or end of life)	14 (12.7%)	(51, 55, 58–60, 68, 71, 74, 77, 86, 93, 96, 97, 103)
Health professional is perceived to lack compassion	11 (10.0%)	(18, 53, 55, 64, 70, 73, 84, 88, 97, 102, 103)
Patients and/or family perceives lack of respect or dignity	9 (8.2%)	(53, 55, 56, 59, 60, 65, 88, 102, 103)
Patients and/or family interest in ALS information limits communication	6 (5.5%)	(19, 63, 66, 69, 82, 87)
Patients and/or family experiences negative emotion when communicating with health professional	4 (3.6%)	(61, 70, 71, 88)
Health professional does not have the information to answer question(s)	6 (5.5%)	(65, 70, 72, 91, 98, 103)
Health professional is reluctant to address end of life	9 (8.2%)	(51, 66, 78, 80, 97–101)
Communication is “forced” by an individual or by disease progression	6 (5.5%)	(39, 70, 91, 95, 97, 104)
**Communication facilitators**	40 (36.4%)	(18, 19, 33, 39, 51, 52, 54–57, 59, 61–63, 68–70, 72, 73, 76, 80, 81, 83–85, 91, 92, 96–98, 103, 106–114)
Health professional is perceived to be friendly or kind	21 (19.1%)	(18, 39, 48, 49, 55–59, 70, 71, 78, 79, 83, 89, 90, 95, 96, 101, 115, 116)
“Sufficient” information given about disease and/or assistive devices	25 (22.7%)	(18, 39, 52, 54, 55, 57, 62, 63, 68–70, 80, 81, 84, 91, 92, 96–98, 103, 111–114, 117)
Access to health professionals, for immediate information needs and ongoing communication or support	16 (14.5%)	(19, 33, 39, 56, 72, 76, 80, 91, 92, 97, 98, 108–110, 112, 114)
Patients and/or family feels satisfied with communication	8 (7.3%)	(18, 61, 63, 73, 85, 91, 92, 103)
Health professional is perceived to be empathetic and/or trustworthy	6 (5.5%)	(55, 59, 80, 81, 97, 103)
Open and/or honest communication	4 (3.6%)	(39, 51, 52, 106)
Patients and/or family feels respected	4 (3.6%)	(39, 70, 91, 107)
**Difficult conversations**	72 (65.5%)	(18, 19, 33, 39, 51, 52, 54–56, 58, 60, 62–70, 72, 73, 75, 76, 78–82, 87, 89, 92, 93, 93–105, 107–111, 114, 118–137)
End-of-life discussion avoidance	38 (34.5%)	(18, 33, 39, 52, 54, 58, 60, 62–64, 68, 69, 75, 76, 82, 93, 94, 96–100, 103, 107, 109, 114, 118–123, 125, 127, 130, 132, 134, 135)
Timing for difficult conversations	39 (35.5%)	(19, 33, 51, 54, 62, 63, 76, 79, 87, 107, 108, 122, 124, 125, 127, 132, 133, 138)
Delivering bad news, health professionals' perspectives	16 (14.5%)	(69, 73, 80, 81, 87, 93, 94, 97, 99–101, 107, 109, 111, 122, 124)
Health professional reluctance to address prognosis	6 (5.5%)	(69, 87, 93, 97, 107, 109)
Clinical education to prepare health professionals for difficult conversations	13 (11.8%)	(39, 55, 67, 73, 78, 80, 81, 89, 97–99, 128, 130)
**Delivering the ALS diagnosis**	25 (22.7%)	(18, 39, 53, 55, 57, 60–63, 69, 73, 80–85, 94, 109, 117, 124, 128, 133, 139, 140)
Method for delivering diagnosis	13 (11.8%)	(39, 55, 57, 60, 62, 63, 73, 80, 81, 85, 124, 128, 139)
Badly communicated diagnosis	11 (10.0%)	(18, 53, 55, 60, 61, 73, 81, 83, 84, 117, 140)
Effective communication of diagnosis	5 (4.5%)	(55, 61, 81, 83, 133)
Skilled delivery of diagnosis is important	5 (4.5%)	(55, 73, 81, 94, 109)
**Functional communication challenges**	45 (40.9%)	(18, 33, 39, 51, 52, 56–58, 63, 64, 67, 71, 76–78, 82, 83, 85, 86, 88, 90, 92–94, 98, 102, 105, 108, 113, 114, 117–119, 127, 132, 134, 138, 139, 141–147)
Severity of communication impairment	27 (24.5%)	(18, 33, 51, 52, 56, 58, 63, 64, 71, 76, 77, 83, 86, 88, 92, 93, 98, 108, 117, 132, 134, 138, 141–145)
Strategies to address speech loss, including AAC	22 (20.0%)	(39, 51, 52, 56–58, 67, 71, 85, 86, 88, 92, 105, 108, 113, 114, 117, 119, 134, 145, 146)
Emotional and social impact of communication challenges	13 (11.8%)	(52, 56, 71, 82, 86, 88, 92, 113, 114, 117, 118, 134, 143)
Impact of devices (e.g., ventilator) on communication	7 (6.4%)	(52, 67, 71, 92, 117, 127, 145)
Effect of AAC on QoL	5 (4.5%)	(52, 67, 71, 88, 94)

Articles that addressed the quality of communication between patients with ALS, families and healthcare professionals noted facilitators (*n* = 40) and barriers (*n* = 57). Facilitators and barriers were characterized not only by communication style, but also by information substance (*what* is communicated) and sufficiency (*enough* information to meet patient need). As might be anticipated, for example, ALS patients and their families valued open and/or “honest” communication with health care professionals (39, 51, 52, 106). In addition, researchers exploring the experiences of ALS caregivers noted that a lack of empathic communication “left the participants feeling shocked, bewildered, angry and devastated” (53). However, this current analysis found that a greater number of articles highlighted the importance of meeting the information needs of patients and families. For example, researchers investigating decisions about life-sustaining treatments reported, “the provision of full information was paramount, which in some cases included providing information in different formats” (54), and neurologists who provided needed or desired information were rated more highly by family caregivers (55). Further, a reviewed study found that ALS patients who “lack communication, information, and clear answers from health providers” experienced “frustration and despair due to a limited life time” (56). Seventeen articles noted seeking information outside the medical system, including online, from interpersonal sources and/or from patient advocacy organizations (18, 51, 54, 57–67, 118, 119, 148).

Of the 72 articles that addressed “difficult conversations,” 38 noted avoidance of end-of-life discussions by ALS patients, their families, and/or healthcare professionals. Twenty-four articles focused on communicating an ALS diagnosis. Articles drawing attention to functional communication challenges related to a motor speech disorder (*n* = 45) primarily highlighted the severity of communication impairments (*n* = 27) and strategies to address speech loss (*n* = 22) (Table 5).

#### Recommendations

Sixty-seven articles made “actionable” recommendations. These were represented by statements of “how” or “what” should be done to improve communication. Recommendations were thematically analyzed. For example, articles with a thematic focus on improving communication processes (actions and steps needed to communicate effectively) included recommendations for the timing of communication about end of life, potential communication mediums (visual, written, web-based), and collaboration between clinicians.

Recommendations in the analyzed articles focused on improving communication processes (*n* = 36), improving communication style (*n* = 21), and improving or changing the content of information communicated to ALS patients and their families (*n* = 21). The 2021 update resulted in one substantial change: 14 articles from 2018 to 2021 recommended ‘more research’ whereas only 4 articles between 1991 and 2017 made this recommendation. Fifteen articles noted a need for communication guidelines or standards, and 15 made a range of recommendations for improving the training of health professionals. A small number of articles specifically recommended shared decision-making (*n* = 5), use of decision-making aids (*n* = 4), and the importance of supporting the patient-caregiver relationship (*n* = 2).

Forty-seven articles directed recommendations to health care professionals; 25 did not specify *who* should carry out the recommended action; and 18 articles made recommendations for researchers. Actionable recommendations were also directed toward health systems (*n* = 9), medical educators (*n* = 8), ALS support organizations (*n* = 3), and family members of patients living with ALS (*n* = 3). Table 6 summarizes analysis of the recommendations found in the included articles.

**Table 6:** Actionable recommendations for improving health communication (*n* = 110).

**Recommendations (number of articles)**	**Target group (number of articles)**	**Examples**
Improve communication practices and/or processes (36)	Health professionals (28), Health systems (8), ALS support organizations (2), Family members (2), Unspecified (3)	*Use of advance directives and collaboration with other related practitioners are recommended to enhance communication linked to psychological care and informed consent*. (76)
Improve communication style (21)	Health professionals (17), Medical education (1), Unspecified (3)	*Use language that patients and their families can understand*. (92)
Improve or amend communication content (21)	Health professionals (14), Family members (1), Unspecified (7)	…*fears of “choking to death” are unwarranted. This information should be available to ALS patients at the time when ventilatory options are discussed*. (89)
More research is needed (18)	Researchers (18)	…*qualitative research in this area is needed to fully understand ACP [advance care planning] preferences and practices among patients*. (129)
Communication guidelines or standards needed (15)	Health professionals (4), ALS support organizations (1)„ Medical education (1), Researchers (1), Unspecified (12)	*More widely available guidelines for the provision of gastrostomy and advice on the best way to impart information to patients and caregivers about gastrostomy and NIV appear to be needed*. (125)
Improve health professionals' training (15)	Medical education (8), Health professionals (4), ALS support organizations (1), Researchers (1), Unspecified (7)	*Medical educators must strive to understand their students' perspectives, adapt their teaching so that they impart compassionate and clinically astute end-of-life care practices*. (78)
Facilitate shared decision-making (5)	Health professionals (5), Health systems (1)	…* the patient and caregiver function as a team, and the caregiver should be included in discussions on treatment and care*. (75)
Use decision-making aids (4)	Health professionals (1), Unspecified (3)	*Our study supports the view that PPC [preferred priorities for care] document should also be offered to MND/ALS patients as a standard of care*. (121)
Improve the patient-caregiver relationship (2)	Health professionals (1), Family members (1)	*Caregivers should take care not only of the patient, but also of themselves, in order to offer adequate support to their loved ones*. (60)

## Discussion

### Main Findings of the Scoping Review

This review identified a limited evidence base and lack of comprehensive recommendations for health communication about end of life with ALS patients. Despite increasing discussion and legalization of voluntary assisted death across jurisdictions (28–30) and its implications for ALS patients (30, 149, 150), there has been only a modest increase over time in empirical investigations of communication about end of life for this population. Moreover, most of the reviewed articles addressed end-of-life communication as a peripheral focus of investigation. In keeping with other studies, this review highlights the need for generic communication skills, including empathy and relationship building (151–154). Findings, however, bring attention to the importance of providing substantive and sufficient disease-related information, including information about symptom management and assistive devices, when discussing end-of-life issues. For people living with ALS, decisions about symptom management, for example dyspnea or dysphagia, may change the natural disease trajectory as technologies introduced for symptom management become life-support technologies (16). Recommendations for communication about end of life with ALS patients primarily target health professionals, providing only general suggestions for improving communication rather than specific, actionable guidelines similar to published guidelines for disclosing an ALS diagnosis (36, 38, 39). The following paragraphs discuss the scope of end-of-life, ALS-focused communication research, perceptions of communication quality, unique challenges for discussions of end of life with ALS patients, and a need for “actionable” communication recommendations that might guide effective communication in clinical practice.

The findings in this review highlight the multidisciplinary nature of health communication research and the concomitant challenge of finding a “home” for ALS-related communication research. Although advances with keyword searching and access to multiple databases mitigate some of these challenges, reviewed articles were published across a wide range of journals and were identified by heterogeneous and poorly standardized database subject headings (155–157). This may introduce challenges for clinicians seeking to find ALS-specific, evidence-based guidance for discussing end of life.

Quality of life for ALS patients and, to a lesser extent, family burden has been widely examined in the ALS literature. These themes appear prominently in the current review, with physical and psychological/emotional domains discussed most frequently as related to one another. For example, articles suggested that planning for end of life was influenced by fear of physical symptoms and of being a burden to loved ones (33, 158, 159). Although communication about the physical aspects of end of life may be viewed as a central task for healthcare professionals, findings suggest, unsurprisingly, that psychological/emotional, social, religious, and even financial factors may also be important aspects of end-of-life communication. In addition to the psychological and emotional toll on ALS patients and their families, research demonstrates substantial emotional burden for healthcare professionals caring for people with terminal neurological disease (41). While patient voices were well represented in the review, articles were less likely to examine the perspectives of healthcare professionals. Given the role that healthcare professionals play in discussing end of life with ALS patients, more research on healthcare professionals' perspectives is needed as a step toward developing guidance for end-of-life communication.

Voluntary assisted death, when discussed, was primarily contextualized within an overarching discussion of end-of-life options. Within the communication subset, very few articles noted assisted death. These findings may be an artifact of the lag between legislative changes and empirical investigation. They may also reflect a tendency toward symptom-driven communication rather than end-of-life discussions that are integrated into clinical care. For example, discussions about end of life may occur in tandem with decisions about initiating, continuing, and/or discontinuing life-sustaining interventions such as mechanical ventilation or enteral feeding tubes. Attitudes toward end-of-life options, including voluntary assisted death, vary across regions and cultures (28, 160, 161). With high mobility within populations, increasing attention to the influence of culture and personal beliefs on advance care planning and decisions for people with ALS (41, 118, 120, 162), and increasing access to voluntary assisted death in many jurisdictions (28, 29), the need for patient-centered evidence and communication guidance is increasingly important for sensitive, effective communication about palliative care and end-of-life options.

The integral role of communication for end-of-life care is documented in the palliative care literature (5, 163, 164). Yet, fewer than half of the selected articles focused explicitly on health communication. These articles—the communication subset—indicate that, despite the importance of online disease-related information (165–168) and support (169, 170) for ALS patients, healthcare professionals are critical information sources for patients and their families. This suggests an important role for professionals both in providing information about end of life, and helping people make sense of information from online sources. Although information needs have been identified as an important domain at the time of the ALS diagnosis (61, 165, 171), research is needed to identify and better understand the information that ALS patients and families want and need to make decisions that influence the disease course and end of life. For example, in contrast to cancer patients, life-sustaining interventions such as nutritional and respiratory support are considered “standard of care” for people living with ALS and are positively associated with improved quality of life (172, 173). Communication about accepting or forgoing such interventions is, therefore, particularly relevant to ALS (and, perhaps, other neurodegenerative disorders). It follows that information about the nuances and practicalities of palliative sedation for the withdrawal of such life-sustaining interventions is important for people with ALS and their families.

Many of the communication challenges identified by this review are not unique to people living with ALS. For example, both this review and the palliative care literature identifies healthcare professionals' reluctance to address prognosis and end-of-life discussion avoidance (41, 115, 174, 175); difficulties identifying appropriate times for conversations about end of life (5, 115, 164); and the changing needs of patients (163). ALS, however, presents additional communication challenges. First, findings demonstrate that disclosing an ALS diagnosis is closely associated with discussions about end of life. Second, throughout the disease course, clinicians must effectively communicate both the chronic and terminal facets of ALS (107). For example, clinicians must guide patients and families through iterative decisions about initiating, maintaining and potentially withdrawing life-sustaining support for nutritional and respiratory needs. Finally, this analysis highlighted functional communication challenges. Almost all ALS patients experience motor speech disorder with disease progression (176, 177). This presents a unique challenge for those seeking to facilitate full and ongoing patient participation in discussions and decisions about end of life.

Thematic analysis makes an important contribution to understanding of the recommendations emerging from the analyzed articles. A small number of themes with specific application were identified (for example, four articles recommended the use of decision aids). However, most recommendations were limited to the specific interventions or gaps in care identified in individual articles and lacked the systematized guidance that is required to operationalize findings for clinical practice. Thematic analysis, however, draws attention to three aspects of communication: processes, style, and the content of communicated information. Findings indicated a primary need for improved communication processes, for example, discussion of end-of-life issues both early and incrementally throughout the disease trajectory (52, 121). Providing substantive information that meets the needs of patients and families was equally important to communication style in the recommendations. These findings draw attention to a need for focused empirical investigation of concrete, evidence-based communication strategies, and the development clinical communication guidelines for discussions about end of life with people living with ALS.

The paucity of focused, end-of-life communication research and the lack of progress in the development of empirically-based communication guidelines for ALS may reflect the tendency for research funding to target marketable interventions and therapies (178, 179). Even among non-profit ALS Societies the overwhelming majority of research funding is directed toward laboratory research, pharmacological interventions, and devices (180–182). Topics such as health communication, which reside at the intersection of Medicine and the Social Sciences, tend to receive limited funding.

### Implications for Research, Policy and Practice

Empirically derived data about end-of-life discussions with ALS patients are primarily embedded in broadly focused investigations. Although there was a small increase in empirical articles, systematic investigation of communication about end of life is limited. The scarcity of research focused on communication, and the increasing number of empirical articles recommending more research in this area, may also reflect a need for proven research methodologies, as well as knowledge and expertise, that will address this evidence gap. Clinicians and researchers need to think of novel, patient-oriented methods to investigate both the communication practices of clinicians and the needs of ALS patients for information about end of life, both at the time of diagnosis and throughout the disease course. Investigations should yield specific, actionable recommendations for translation into policy and practice. This will provide a foundation for developing guidelines supporting end-of-life communication between health professionals and ALS patients and their families.

As discussed, findings may reflect policies and practices that direct research funding to marketable interventions and therapies. Despite the importance of these activities, communication is critical to the clinical management of ALS. Policies that promote the funding of communication research will provide a foundation for developing an evidence-base for compassionate, effective, and ethical communication about end of life, as well as evidence-based communication training in educational institutions and via continuing education for health professionals who care for ALS patients.

Finally, this review has implications for medical practitioners. The wide range of journals publishing research in this area of investigation may compromise access for practicing clinicians. Highly ranked journals that are specific to neurology and palliative care should seek to provide a home for this body for research that represents both the science and “art” of medicine. Further, this review draws attention to communication quality as mediated not only by core communication skills, but also by information substance and sufficiency. While emotional connection is important, the clinical expertise and information communicated by health professionals builds trust and “ownership” of care decisions (181, 182).

Clinical discussion of issues related to end of life has substantial impact on care and facilitates compliance with patients' wishes (169). Actionable recommendations and guidance are needed to support clinicians caring for patients with ALS. This is particularly important because ALS specialists and multidisciplinary ALS clinics are concentrated in large urban centers that may become inaccessible with disease progression. ALS patients frequently begin to rely on support from palliative and community physicians at a time when they need expert and nuanced information. Developing a strong empirical foundation and end-of-life communication guidance will support both specialists and non-specialists as they iteratively discuss life-sustaining therapies and end-of-life issues with ALS patients and their families.

### Strengths and Limitations

This investigation followed standard methodological recommendations for scoping reviews, as well as Levac et al.'s recommendations to include both numerical summary analysis and qualitative content analytical techniques when summarizing and reporting results (45, 47, 48). Recommendations to consider the review's implications within the broader contexts of research, policy and practice were also followed (47).

A primary strength of this review is the focus on primary research articles. Although many review and commentary articles that may provide insight into end-of-life communication were excluded, this review makes an important contribution by documenting the paucity of empirical evidence in this area of investigation. Better understanding of the scope and nature of the evidence, both quantitative and qualitative, provides a starting point for systematically addressing evidence gaps. Further, because the review included all empirical articles available in the databases without time restriction, these data meet the study objectives and provide an overarching view of this research area.

There are limitations to this review. Critical appraisal of articles was limited to the application of inclusion/exclusion criteria. For example, articles that did not report primary quantitative and/or qualitative empirical data were excluded. The rigor of research processes within individual studies was not evaluated.Restriction to articles published in the English language presents another limitation. Communication, particularly about end of life, is rooted in cultural expectations and practice. Some of the review's outcomes could, therefore, be an artifact of the language restriction.

Finally, despite the profound impact of legislative changes on end-of-life decisions for ALS patients (68, 122, 150, 183), the heterogenous methods used in the fields of Medicine and Law presented a methodological limitation. Whereas empirical data are central to high quality evidence in scientific fields such as Neurology (184), legal research focuses on doctrinal and comparative analysis of authoritative texts with reasoning and conceptual analysis as an indicator of quality (116, 185). Therefore, articles published in legal journals did not meet study inclusion criteria. Although scoping review methodology facilitates review of articles with varying research designs (45), further methodological development is needed to facilitate review and analysis of high-quality evidence emerging from the disparate research traditions of Medicine and Law.

## Conclusion

This review demonstrates a small increase in empirical articles discussing end-of-life communication with people living with ALS (1991–May 2021). Most reviewed articles were published in clinical neurology journals. However, the articles were published in large number of different journals with only a small number published in each. Overall, communication about the end of life remains a peripheral part of more broadly focused investigations. This review found that generic communication skills, such as expressing empathy, were important; however, information substance and sufficiency was central to high quality, effective health communication. Recommendations for clinical communication focused on communication processes, style, and content, but lacked systematic guidance. Despite the absence of communication guidelines for end of life, practice recommendations for the management of ALS encourage clinicians to discuss life-sustaining therapies and end of life with ALS patients (16, 36, 37). This review supports these recommendations by highlighting the need for focused, empirical investigation of best practices for end-of-life communication. This will provide a foundation for evidence-based, ALS-specific guidelines for communication about the end of life. Particularly with increasing options at end of life, actionable recommendations and guidance is needed to support ALS clinicians as they iteratively discuss life-sustaining therapies and end-of-life issues with patients and families.

## Data Availability Statement

The raw data supporting the conclusions of this article will be made available by the authors, without undue reservation.

## Author Contributions

TB and WJ conceived the original study and developed the design and methods. SC prepared and executed the search strategy. WL and SG screened studies for inclusion and carried out data extraction for quantitative analysis and qualitative data analysis. SG and WL conducted the synthesis with input from TB and WJ. SG drafted the manuscript with contribution from WL, TB, and WJ. All authors contributed to the article and approved the submitted version.

## Conflict of Interest

The authors declare that the research was conducted in the absence of any commercial or financial relationships that could be construed as a potential conflict of interest.

## Publisher's Note

All claims expressed in this article are solely those of the authors and do not necessarily represent those of their affiliated organizations, or those of the publisher, the editors and the reviewers. Any product that may be evaluated in this article, or claim that may be made by its manufacturer, is not guaranteed or endorsed by the publisher.
