# Supplementary material for: Communication About End of Life for Patients Living With Amyotrophic Lateral Sclerosis: A Scoping Review of the Empirical Evidence
Source: Front Neurol. 2021 Aug 4;12:683197. doi: 10.3389/fneur.2021.683197 (PMC8371472; doi:10.3389/fneur.2021.683197)
Supplement: Supplementary file 1 [file Table_1.pdf]

Communication about end of life for patients living with amyotrophic lateral sclerosis: A scoping review of the empirical evidence

Supplementary file 1: Sample search, Medline (Ovid)

Ovid MEDLINE(R) ALL <1946 to May 03, 2021> Searched May 05, 2021

#	Search Statement	Results
1	exp amyotrophic lateral sclerosis/ or amyotrophic lateral sclero*.mp. or (Kathleen Farrell or Brittany Maynard or Sue Rodriguez or Kay Carter or Gloria Taylor or als).ti,ab. [mp=title, abstract, original title, name of substance word, subject heading word, floating sub-heading word, keyword heading word, organism supplementary concept word, protocol supplementary concept word, rare disease supplementary concept word, unique identifier, synonyms]	45103
2	((guam or gehrig* or charcot*) adj2 disease*).mp. [mp=title, abstract, original title, name of substance word, subject heading word, floating sub-heading word, keyword heading word, organism supplementary concept word, protocol supplementary concept word, rare disease supplementary concept word, unique identifier, synonyms]	532
3	parkinsonism dementia.mp.	533
4	lou gherig*.mp.	1
5	lou gehrig*.mp.	251
6	(guam adj1 form).mp. [mp=title, abstract, original title, name of substance word, subject heading word, floating sub-heading word, keyword heading word, organism supplementary concept word, protocol supplementary concept word, rare disease supplementary concept word, unique identifier, synonyms]	0
7	1 or 2 or 3 or 4 or 5 or 6	45510
8	exp Attitude to Death/ or attitude* to death*.mp.	16594
9	exp Terminal Care/	52662
10	exp Palliative Care/	56093
11	palliat*.mp.	102426
12	exp Terminally Ill/ or exp Mortality/ [mp=title, abstract, original title, name of substance word, subject heading word, floating sub-heading word, keyword heading word, organism supplementary concept word, protocol supplementary	404640

	concept word, rare disease supplementary concept word, unique identifier, synonyms]	
13	(terminal adj1 (ill* or care or carer* or caring or wishes or preference*)).mp. [mp=title, abstract, original title, name of substance word, subject heading word, floating sub-heading word, keyword heading word, organism supplementary concept word, protocol supplementary concept word, rare disease supplementary concept word, unique identifier, synonyms]	31503
14	(end of life or death or dying or mortality or terminally ill).mp.	884497
15	((critical* adj1 ill*) or eol).mp.	67759
16	exp Death/	152177
17	exp Thanatology/ or mors.ti,ab.	1020
18	thanatolog*.mp.	617
19	exp Bereavement/	13718
20	bereav*.mp.	10494
21	grief.mp. or exp Grief/	12817
22	grieving.mp.	1626
23	exp Euthanasia/ or exp Right to Die/ or Resuscitation Orders/ or exp Advance Directives/ or Advance Care Planning/ or exp Withholding Treatment/ or ("right to die" or "allowing to die" or advance* directive* or advance* care plan* or living will* or personal directive* or euthan* or mercy killing or self deliverance or exit bag or debreather or accelerated death or "do not resuscitate" or DNR).mp. or (Brittany Maynard or Sue Rodriguez or Nancy Cruzan or Kay Carter or Gloria Taylor or Donald Low or Robert Latimer or Kevorkian or Thomas Youk).ti,ab. or ((suicid* or die) adj2 (hasten* or voluntary or request* or aided or aiding or assist* or prefer* or dignity or plan* or dignif* or choice or decision)).mp.	73396
24	Hospices/	5119
25	hospice*.mp.	18054
26	8 or 9 or 10 or 11 or 12 or 13 or 14 or 15 or 16 or 17 or 18 or 19 or 20 or 21 or 22 or 23 or 24 or 25	1413192
27	7 and 26	5905

28	exp Health Communication/ or communicat*.mp.	444455
29	exp Physician-Patient Relations/ or exp Truth Disclosure/ or truth disclose*.mp.	85973
30	((difficult or tough or hard or delicate or trying) adj1 (conversation or talk or discussion)).tw.	88
31	((break* or disclos* or deliver* or bad) adj2 news).mp. [mp=title, abstract, original title, name of substance word, subject heading word, floating sub-heading word, keyword heading word, organism supplementary concept word, protocol supplementary concept word, rare disease supplementary concept word, unique identifier, synonyms]	2620
32	exp Patient Preference/ or exp Patient Satisfaction/ or ((patient* or family or families) adj2 (preference* or satisfaction* or involve* or participat*)).mp.	207678
33	exp Patient Participation/ or (informed consent* or patient decision making).mp. or exp Informed Consent/ [mp=title, abstract, original title, name of substance word, subject heading word, floating sub-heading word, keyword heading word, organism supplementary concept word, protocol supplementary concept word, rare disease supplementary concept word, unique identifier, synonyms]	95773
34	28 or 29 or 30 or 31 or 32 or 33	752174
35	27 and 34	328
36	limit 35 to animals	43
37	35 not 36	285
38	remove duplicates from 37	284
