# Supplementary material for: Communication About End of Life for Patients Living With Amyotrophic Lateral Sclerosis: A Scoping Review of the Empirical Evidence
Source: Front Neurol. 2021 Aug 4;12:683197. doi: 10.3389/fneur.2021.683197 (PMC8371472; doi:10.3389/fneur.2021.683197)
Supplement: Supplementary file 2 [file Table_2.pdf]

Communication about end of life for patients living with amyotrophic lateral sclerosis: A scoping review of the empirical evidence

Supplementary file 2: Included studies

Author(s)	Year	Title	Journal
Abrahao, A., Downar, J., Pinto, H., Dupré, N., Izenberg, A., Kingston, W., Korngut, L., O'Connell, C., Petrescu, N., Shoesmith, C., Tandon, A., Vargas-Santos, A. B., and Zinman, L.	2016	Physician-assisted death: A Canada-wide survey of ALS health care providers	Neurology
Abuzinadah, A. R., Al Shareef, A. A., AlKutbi, A., Bamaga, A. K., Alshehri, A., Algahtani, H., Cupler, E., and Alanazy, M. H.	2020	Amyotrophic lateral sclerosis care in Saudi Arabia: A survey of providers' perceptions	Brain and Behaviour
Achille, M A., and Ogloff, J. R. P.	2003	Attitudes toward and desire for assisted suicide among persons with amyotrophic lateral sclerosis	Omega: Journal Of Death And Dying
Adelman, E. E., Albert, S. M., Rabkin, J. G., Del Bene, M. L., Tider, T., and O'Sullivan, I.	2004	Disparities in perceptions of distress and burden in ALS patients and family caregivers	Neurology
Ahlberg, E. E., and Axelsson, B.	2021	End-of-life care in amyotrophic lateral sclerosis: A comparative registry study	Acta neurologica Scandinavica
Albert, S. M., Murphy, P. L., Del Bene, M. L., and Rowland, L. P.	1999	Prospective study of palliative care in ALS: Choice, timing, outcomes	Journal Of The Neurological Sciences
Albert, S. M., Murphy, P. L., Del Bene, M., Rowland, L. P., and Mitsumoto, H.	2001	Incidence and predictors of PEG placement in ALSMND	Journal Of The Neurological Sciences
Albert, S. M., Rabkin, J. G., Del Bene, M. L., Tider, T., O'Sullivan, I., Rowland, L. P., and Mitsumoto, H.	2005	Wish to die in end-stage ALS	Neurology
Albert, S. M., Wasner, M., Tider, T., Drory, V. E., and Borasio, G. D.	2007	Cross-cultural variation in mental health at end of life in patients with ALS	Neurology
Albert, S. M., Whitaker, A., Rabkin, J. G., del Bene, M., Tider, T., O'Sullivan, I., and Mitsumoto, H.	2009	Medical and Supportive Care Among People with ALS in the Months Before Death or Tracheostomy	Journal Of Pain And Symptom Management

Andersen, P. M., Kuzma-Kozakiewicz, M., Keller, J., Aho-Oezhan, H. E. A., Ciecwierska, K., Szejko, N., Vázquez, C., Böhm, S., Badura-Lotter, G., Meyer, T., Petri, S., Linse, K., Hermann, A., Semb, O., Stenberg, E., Nackberg, S., Dorst, J., Uttner, I., Häggström, A. - C., Ludolph, A. C., and Lulé, D.	2018	Therapeutic decisions in ALS patients: cross-cultural differences and clinical implications	Journal of Neurology
Ando, H., Cousins, R., and Young, C.A.	2019	Exploring and Addressing 'Concerns' for Significant Others to Extend the Understanding of Quality of Life With Amyotrophic Lateral Sclerosis: A Qualitative Study	Journal of Central Nervous System Disease
Ang, K., Umapathi, T., Tong, J., Ng, J., Tseng, L. J., and Woo, I. M. H.	2015	Healthcare needs of patients with amyotrophic lateral sclerosis (ALS) in Singapore: A patient-centred qualitative study from multiple perspectives	Journal Of Palliative Care
Ansari, S., Bromberg, M. B., and Gibson, S.B.	2017	Physician perceptions about living organ donation in patients with Amyotrophic Lateral Sclerosis	Clinical Neurology And Neurosurgery
Aoun, S. M., Breen, L.J., Edis, R., Henderson, R. D., Oliver, D., Harris, R., Howting, D., O'Connor, M., and Birks, C.	2016	Breaking the news of a diagnosis of motor neurone disease: A national survey of neurologists' perspectives	Journal Of The Neurological Sciences
Aoun, S.M., Breen, L.J., Howting, D., Edis, R., Oliver, D., Henderson, R., O'Connor, M., Harris, R., and Birks, C.	2016	Receiving the news of a diagnosis of motor neuron disease: What does it take to make it better?	Amyotrophic Lateral Sclerosis And Frontotemporal Degeneration
Aoun, S. M., Breen, L. J., Oliver, D., Henderson, R.D., Edis, R., O'Connor, M., Howting, D., Harris, R., and Birks, C.	2017	Family carers' experiences of receiving the news of a diagnosis of Motor Neurone Disease: A national survey	Journal Of The Neurological Sciences
Aoun, S. M., Chochinov, H. M., and Kristjanson, L. J.	2015	Dignity Therapy for People with Motor Neuron Disease and Their Family Caregivers: A Feasibility Study	Journal Of Palliative Medicine
Aoun, S. M., Connors, S. L., Priddis, L., Breen, L. J., and Colyer, S.	2012	Motor Neurone Disease family carers' experiences of caring, palliative care and bereavement: An exploratory qualitative study	Palliative Medicine

Astrow, A. B., Sood, J. R., Nolan, M. T., Terry, P. B., Clawson, L., Hughes, M., and Sulmasy, D. P.	2008	Decision-making in patients with advanced cancer compared with amyotrophic lateral sclerosis	Journal Of Medical Ethics: Journal Of The Institute Of Medical Ethics
Bach, J. R.	1993	Amyotrophic lateral sclerosis: communication status and survival with ventilatory support	American Journal Of Physical Medicine And Rehabilitation
Bae, J. S., Hong, Y. - H., Baek, W., Sohn, E. H., Cho, J. - Y., Kim, B. - J., Kim, S. H., and Korean ALS/MND Research Group	2012	Current Status of the Diagnosis and Management of Amyotrophic Lateral Sclerosis in Korea: A Multi-Center Cross-Sectional Study	Journal Of Clinical Neurology
Bahus, M. K., Steen, P. A., and Forde, R.	2012	Law, ethics and clinical judgment in end-of-life decisions-How do Norwegian doctors think?	Resuscitation
Baxter, S. K., Baird, W. O., Thompson, S., Bianchi, S. M., Walters, S. J., Lee, E., Ahmedzai, S. H., Proctor, A., Shaw, P. J., and McDermott, C. J.	2013	The use of non-invasive ventilation at end of life in patients with motor neurone disease: A qualitative exploration of family carer and health professional experiences	Palliative Medicine
Beghi, E., Logroscino, G., Micheli, A., Millul, A., Perini, M., Riva, R., Salmoiraghi, F., Vitelli, E., and Italian Registry Study Group	2001	Validity of hospital discharge diagnoses for the assessment of the prevalence and incidence of amyotrophic lateral sclerosis	Amyotrophic Lateral Sclerosis And Other Motor Neuron Disorders
Bello-Haas, V. D., Andrews-Hinders, D., Bocian, J., Mascha, E., Wheeler, T., and Mitsumoto, H.	2000	Spiritual well-being of the individual with amyotrophic lateral sclerosis	Amyotrophic Lateral Sclerosis
Bentley, B., O'Connor, M., Kane, R., Breen L. J.	2014	Feasibility, acceptability, and potential effectiveness of dignity therapy for people with motor neurone disease	PLoS One
Bentley, B., O'Connor, M., Breen, L. J., and Kane, R.	2014	Feasibility, acceptability and potential effectiveness of dignity therapy for family carers of people with motor neurone disease	Bmc Palliative Care
Blanke, C., LeBlanc, M., Hershman, D., Ellis, L., and Meyskens, F.	2017	Characterizing 18 years of the death with dignity act in Oregon	JAMA Oncology
Bolmsjö, I., and Hermérn, G.	2003	Conflicts of interest: Experiences of close relatives of patients suffering from amyotrophic lateral sclerosis	Nursing Ethics

Bolmsjö, I.	2001	Existential issues in palliative care: interviews of patients with amyotrophic lateral sclerosis	Journal Of Palliative Medicine
Bolmsjö, I., and Hermérn, G.	2001	Interviews with patients, family, and caregivers in amyotrophic lateral sclerosis: comparing needs	Journal Of Palliative Care
Borasio, G. D., Shaw, P. J., Hardiman, O., Ludolph, A. C., Sales Luis, M. L., and Silani, V.	2001	Standards of palliative care for patients with amyotrophic lateral sclerosis: Results of a European survey	Amyotrophic Lateral Sclerosis And Other Motor Neuron Disorders
Bourke, S. C., Bullock, R. E., Williams, T. L., Shaw, P. J., and Gibson, G. J.	2003	Noninvasive ventilation in ALS: Indications and effect on quality of life	Neurology
Bradley, W. G., Anderson, F., Bromberg, M., Gutmann, L., Harati, Y., Ross, M., and Miller, R. G.	2001	Current management of ALS - Comparison of the ALS CARE Database and the AAN Practice Parameter	Neurology
Bromberg, M. B., and Forshew, D. A.	2002	Comparison of instruments addressing quality of life in patients with ALS and their caregivers	Neurology
Bromberg, M. B., Forshew, D. A., Iaderosa, S., and McDonald, E. R.	1996	Ventilator dependency in ALS: management, disease progression, and issues of coping	Journal Of Neurologic Rehabilitation
Brown, J., and Addington-Hall, J.	2008	How people with motor neurone disease talk about living with their illness: A narrative study	Journal Of Advanced Nursing
Burchardi, N., Rauprich, O., Hecht, M., Beck, M., and Vollmann, J.	2005	Discussing living wills. A qualitative study of a German sample of neurologists and ALS patients	Journal Of The Neurological Sciences
Burkhardt, C., Neuwirth, C., Sommacal, A., Andersen, P.M., and Weber, M.	2017	Is survival improved by the use of NIV and PEG in amyotrophic lateral sclerosis (ALS)? A post-mortem study of 80 ALS patients	PLoS One
Bužgová, R., Kozáková, R., and Juríčková, L.	2019	The Unmet Needs of Patients With Progressive Neurological Diseases in the Czech Republic: A Qualitative Study	Journal of Palliative Care
Bužgová, R., Kozáková, R., and Juríčková, L.	2019	The unmet needs of family members of patients with	PloS One

		progressive neurological disease in the Czech Republic	
Carver, A. C., Vickrey, B. G., Bernat, J. L., Keran, C., Ringel, S. P., and Foley, K. M.	1999	End-of-life care: A survey of US neurologists' attitudes, behavior, and knowledge	Neurology
Cazzolli, P. A., and Oppenheimer, E. A.	1996	Home mechanical ventilation for amyotrophic lateral sclerosis: Nasal compared to tracheostomy-intermittent positive pressure ventilation	Journal Of The Neurological Sciences
Cheng, H. W. B., Chan, O. M. I., Chan, C. H. R., Chan, W. H., Fung, K. S., and Wong, K. Y.	2018	End-of-life characteristics and palliative care provision for patients with motor neuron disease	The American Journal of Hospice & Palliative Care
Cheung, K.-C., Lau, V. W.-K., Un, K.-C., Wong, M.-S., and Chan, K.-Y.	2018	Advance care planning for patients with advanced neurology diseases	Annals of Palliative Medicine
Chhetri, S. K., Bradley, B. F., Callagher, P., Addison-Jones, R., Bennett, W., Gardham, J., Parkes, A., Lea, R. W., and Majeed, T.	2015	Choosing the place of death: Empowering motor neurone disease/amyotrophic lateral sclerosis patients in end-of-life care decision making	Palliative Medicine
Chiò, A., and Silani, V.	2001	Amyotrophic lateral sclerosis care in Italy: A nationwide study in neurological centers	Journal Of The Neurological Sciences
Chiò, A., Gauthier, A., Vignola, A., Calvo, A., Ghiglione, P., Cavallo, E., Terreni, A. A., and Mutani, R.	2006	Caregiver time use in ALS	Neurology
Chiò, A., Hammond, E. R., Mora, G., Bonito, V., and Filippini, G.	2015	Development and evaluation of a clinical staging system for amyotrophic lateral sclerosis	Journal Of Neurology, Neurosurgery And Psychiatry
Chiò, A., Montuschi, A., Cammarosano, S., De Mercanti, S., Cavallo, E., Ilardi, A., Ghiglione, P., Mutani, R., and Calvo, A.	2008	ALS patients and caregivers communication preferences and information seeking behaviour	European Journal Of Neurology
Chochinov, H. M., Johnston, W., McClement, S. E., Hack, T. F., Dufault, B., Enns, M., Thompson, G., Harlos, M., Damant, R. W., Ramsey, C. D., Davison, S., Zacharias, J., Milke, D., Strang, D., Campbell-Enns, H. J., and Kredentser, M. S.	2016	Dignity and distress towards the end of life across four non-cancer populations	PLoS One

Christodoulou, G., Goetz, R., Ogino, M., Mitsumoto, H., and Rabkin, J.	2016	Opinions of Japanese and American ALS caregivers regarding tracheostomy with invasive ventilation (TIV)	Amyotrophic Lateral Sclerosis And Frontotemporal Degeneration
Cipolletta, S., and Amicucci, L.	2015	The family experience of living with a person with amyotrophic lateral sclerosis: a qualitative study	International Journal Of Psychology
Cipolletta, S., and Reggiani, M.	2021	End-of-life care after the legal introduction of advance directives: A qualitative study involving healthcare professionals and family caregivers of patients with amyotrophic lateral sclerosis	Palliative Medicine
Clabburn, O., Knighting, K., Jack, B.A., and O’Brien, M. R.	2019	The use of digital legacies with people affected by motor neurone disease for continuing bonds: An interpretative phenomenological analysis study	Palliative Medicine
Clarke, S., Hickey, A., O'Boyle, C., and Hardiman, O.	2001	Assessing individual quality of life in amyotrophic lateral sclerosis	Quality Of Life Research
Costa, T. D. de C., Alves, A. M. P. de M., Costa, E. de O., Acioly, C. M. C., Batista, P. S. de S.	2020	Palliative care to patients with amyotrophic lateral sclerosis: experiences of physiotherapists in a hospital setting	Revista de Pesquisa: Cuidado e Fundamental
Craig, A., and Dzeng, E.	2018	How should physicians care of dying patient with amyotrophic lateral sclerosis?	AMA Journal of Ethics
Creemers, H., Beelen, A., Grupstra, H., Nollet, F., and Van den Berg, L. H.	2014	The provision of assistive devices and home adaptations to patients with ALS in the Netherlands: Patients' perspectives	Amyotrophic Lateral Sclerosis And Frontotemporal Degeneration
Dreyer, P. S., Felding, M., Klitnaes, C. S., and Lorenzen, C. K.	2012	Withdrawal of Invasive Home Mechanical Ventilation in Patients with Advanced Amyotrophic Lateral Sclerosis: Ten Years of Danish Experience	Journal Of Palliative Medicine
Escarrabill, J., Vianello, A., Farrero, E., Ambrosino, N., Llorens, J. M., and Vitacca, M.	2014	Place of death in patients with amyotrophic lateral sclerosis	Revista Portuguesa De Pneumologia

Esposito, S. J., Mitsumoto, H., and Shanks, M.	2000	Use of palatal lift and palatal augmentation prostheses to improve dysarthria in patients with amyotrophic lateral sclerosis: a case series	Journal Of Prosthetic Dentistry
Fanos, J. H., Gelinas, D. F., and Miller, R. G.	2004	"You have shown me my end":Attitudes toward presymptomatic testing for familial amyotrophic lateral sclerosis	American Journal Of Medical Genetics
Fegg, M. J., Kögler, M., Brandstätter, M., Jox, R., Anneser, J., Haarmann-Doetkotte, S., Wasner, M., and Borasio, G. D.	2010	Meaning in life in patients with amyotrophic lateral sclerosis	Amyotrophic Lateral Sclerosis
Fischer, S., Huber, C. A., Imhof, L., Imhof, R. M., Furter, M., Ziegler, S. J., and Bosshard, G.	2008	Suicide assisted by two Swiss right-to-die organisations	Journal Of Medical Ethics: Journal Of The Institute Of Medical Ethics
Flaherty-Craig, C., Eslinger, P., Stephens, B., and Simmons, Z.	2006	A rapid screening battery to identify frontal dysfunction in patients with ALS	Neurology
Foley, G., Timonen, V., and Hardiman, O.	2014	Acceptance and decision making in amyotrophic lateral sclerosis from a life-course perspective	Qualitative Health Research
Foley, G., Timonen, V., and Hardiman, O.	2014	Understanding psycho-social processes underpinning engagement with services in motor neurone disease: A qualitative study	Palliative Medicine
Funke, A., Spittel, S., Grehl, T., Grosskreutz, J., Kettemann, D., Petri, S., Weyen, U., Weydt, P., Dorst, J., Ludolph, A.C., Baum, P., Oberstadt, M., Jordan, B., Hermann, A., Wolf, J., Boentert, M., Walter, B., Gajewski, N., Maier, A., Münch, C., and Meyer, T.	2018	Provision of assistive technology devices among people with ALS in Germany: a platform-case management approach	Amyotrophic Lateral Sclerosis And Frontotemporal Degeneration
Gale, C.	2015	Assisting patients with motor neurone disease to make decisions about their care	International Journal Of Palliative Nursing
Ganzini, L., Goy, E. R., Dobscha, S. K., and Prigerson, H.	2009	Mental Health Outcomes of Family Members of Oregonians Who Request Physician Aid in Dying	Journal Of Pain And Symptom Management

Ganzini, L., Johnston, W. S., and Silveira, M. J.	2002	The final month of life in patients with ALS	Neurology
Ganzini, L., Johnston, W. S., McFarland, B. H., Tolle, S. W., and Lee, M. A.	1998	Attitudes of patients with amyotrophic lateral sclerosis and their care givers toward assisted suicide	New England Journal Of Medicine
Ganzini, L., Silveira, M. J., and Johnston, W. S.	2002	Predictors and correlates of interest in assisted suicide in the final month of life among ALS patients in Oregon and Washington	Journal Of Pain And Symptom Management
Gauthier, A., Vignola, A., Calvo, A., Cavallo, E., Moglia, C., Sellitti, L., Mutani, R., and Chiò, A.	2007	A longitudinal study on quality of life and depression in ALS patient-caregiver couples	Neurology
Gelinas, D. F., O'Connor, P., and Miller, R. G.	1998	Quality of life for ventilator-dependent ALS patients and their caregivers	Journal Of The Neurological Sciences
Gofton, T. E., Chum, M., Schulz, V., Gofton, B. T., Sarpal, A., and Watling, C.	2018	Challenges facing palliative neurology practice: A qualitative analysis	Journal of the Neurological Sciences
Goldstein, L. H., Adamson, M., Jeffrey, L., Down, K., Barby, T., Wilson, C., and Leigh, P. N.	1998	The psychological impact of MND on patients and carers	Journal Of The Neurological Sciences
Goranson, A., Ritter, R. S., Waytz, A., Norton, M. I., and Gray, K.	2017	Dying Is Unexpectedly Positive	Psychological Science
Greenaway, L. P., Martin, N. H., Lawrence, V., Janssen, A., Al-Chalabi, A., Leigh, P. N., and Goldstein, L. H.	2015	Accepting or declining non-invasive ventilation or gastrostomy in amyotrophic lateral sclerosis: patients' perspectives	Journal Of Neurology
Hack, T. F., McClement, S. E., Chochinov, H. M., Dufault, B., Johnston, W., Enns, M. W., Thompson, G. N., Harlos, M., Damant, R. W., Ramsey, C. D., Davison, S. N., Zacharias, J., Strang, D., and Campbell-Enns, H. J.	2018	Assessing symptoms, concerns, and quality of life in noncancer patients at end of life: How concordant are patients and family proxy members?	Journal Of Pain And Symptom Management
Hartzfeld, D. E. H., Siddique, N., Victorson, D., O'Neill, S., Kinsley, L., and Siddique, T.	2015	Reproductive decision-making among individuals at risk for familial amyotrophic lateral sclerosis	Amyotrophic Lateral Sclerosis And Frontotemporal Degeneration

Hayashi, H., and Oppenheimer, E. A.	2003	ALS patients on TPPV: totally locked-in state, neurologic findings and ethical implications	Neurology
Hecht, M. J., Graesel, E., Tigges, S., Hillemacher, T., Winterholler, M., Hilz, M. J., Heuss, D., and Neundorfer, B.	2003	Burden of care in amyotrophic lateral sclerosis	Palliative Medicine
Hecht, M., Hillemacher, T., Gräsel, E., Tigges, S., Winterholler, M., Heuss, D., Hilz, M. J., and Neundörfer, B.	2002	Subjective experience and coping in ALS	Amyotrophic Lateral Sclerosis And Other Motor Neuron Disorders
Hedberg, K., and New, C.	2017	Oregon's death with dignity act: 20 years of experience to inform the debate	Annals Of Internal Medicine
Helleman, J., Eenennaam, R. V., Kruitwagen, E. T., Kruithof, W. J., Slappendel, M. J., Berg, L. H. V. D., Visser-Meily, J. M. A., and Beelen, A.	2020	Telehealth as part of specialized ALS care: feasibility and user experiences with “ALS home-monitoring and coaching”	Amyotrophic Lateral Sclerosis And Frontotemporal Degeneration
Hicks, F., and Corcoran, G.	1993	Should hospices offer respite admissions to patients with motor neurone disease?	Palliative Medicine
Ho, G. W. K., Skaggs, L., Yenokyan, G., Kellogg, A., Johnson, J. A., Lee, M. C., Heinze, K., Hughes, M. T., Sulmasy, D. P., Kub, J., Terry, P. B., Astrow, A. B., Zheng, J., Lehmann, L. S., and Nolan, M. T.	2017	Patient and caregiver characteristics related to completion of advance directives in terminally ill patients	Palliative And Supportive Care
Hogden, A., Greenfield, D., Nugus, P., and Kiernan, M. C.	2015	Development of a model to guide decision making in amyotrophic lateral sclerosis multidisciplinary care	Health Expectations
Hogden, A., Greenfield, D., Nugus, P., and Kiernan, M. C.	2012	Engaging in patient decision-making in multidisciplinary care for amyotrophic lateral sclerosis: the views of health professionals	Patient Preference And Adherence
Hogden, A., Greenfield, D., Nugus, P., and Kiernan, M. C.	2012	What influences patient decision-making in amyotrophic lateral sclerosis multidisciplinary care? A study of patient perspectives	Patient Preference And Adherence

Hubbard, G., McLachlan, K., Forbat, L., and Munday, D.	2012	Recognition by family members that relatives with neurodegenerative disease are likely to die within a year: A meta-ethnography	Palliative Medicine
Jeppesen, J., Rahbek, J., Gredal, O., and Hansen, H. P.	2014	How Narrative Journalistic Stories Can Communicate the Individual's Challenges of Daily Living with Amyotrophic Lateral Sclerosis	Patient-Patient Centered Outcomes Research
Johnson, J. O., Sulmasy, D. P., and Nolan, M. T.	2007	Patients' experiences of being a burden on family in terminal illness	Journal Of Hospice And Palliative Nursing
Kühnlein, P., Kübler, A., Raubold, S., Worrell, M., Kurt, A., Gdynia, H. J., Sperfeld, A. D., and Ludolph, A. C.	2008	Palliative care and circumstances of dying in German ALS patients using non-invasive ventilation	Amyotrophic Lateral Sclerosis
Kang, S.-C., Hwang, S.-J., Wu, P.-YY., and Tsai, C.-P.	2013	The utilization of hospice care among patients with motor neuron diseases: The experience in Taiwan from 2005 to 2010	Journal Of The Chinese Medical Association
Kaub-Wittemer, D., Von Steinbuchel, N., Wasner, M., Laier-Groeneveld, G., and Borasio, G. D.	2003	Quality of life and psychosocial issues in ventilated patients with amyotrophic lateral sclerosis and their caregivers	Journal Of Pain And Symptom Management
Kenny, R. W.	2002	The death of Loving: maternal identity as moral constraint in a narrative testimonial advocating physician assisted suicide	Health Communication
Kleinbub, J. R., Palmieri, A., Broggio, A., Pagnini, F., Benelli, E., Sambin, M., and Soraru, G.	2015	Hypnosis-based psychodynamic treatment in ALS: a longitudinal study on patients and their caregivers	Frontiers In Psychology
Koerner, S., Kollewe, K., Abdulla, S., Zapf, A., Dengler, R., and Petri, S.	2015	Interaction of physical function, quality of life and depression in Amyotrophic lateral sclerosis: characterization of a large patient cohort	BMC Neurology
Krivickas, L. S., Shockley, L., and Mitsumoto, H.	1997	Home care of patients with amyotrophic lateral sclerosis (ALS)	Journal Of The Neurological Sciences
Kubler, A., Winter, S., Ludolph, A. C., Hautzinger, M., and Birbaumer, N.	2005	Severity of depressive symptoms and quality of life in patients with amyotrophic lateral sclerosis	Neurorehabilitation And Neural Repair

Kukulka, K., Washington, K. T., Govindarajan, R., and Mehr, D. R.,	2019	Kukulka, K., Washington, K.T., Govindarajan, R., Mehr, D.R., 2019. Stakeholder Perspectives on the Biopsychosocial and Spiritual Realities of Living With ALS: Implications for Palliative Care Teams	American Journal of Hospice & Palliative Medicine
Kurisaki, R., Yamashita, S., Sakamoto, T., Maruyoshi, N., Uekawa, K., Uchino, M., and Ando, Y.	2014	Decision making of amyotrophic lateral sclerosis patients on noninvasive ventilation to receive tracheostomy positive pressure ventilation	Clinical Neurology And Neurosurgery
Kuzma-Kozakiewicz, M., Andersen, P. M., Ciecwierska, K., Vázquez, C., Helczyk, O., Loose, M., Uttner, I., and Lulé, D.	2019	An observational study on quality of life and preferences to sustain life in locked-in state	Neurology
Larsson, B. J., Fröjd, C., Nordin, K., and Nygren, I.	2015	Relatives of patients with amyotrophic lateral sclerosis: Their experience of care and support	Palliative And Supportive Care
Lechtzin, N., Wiener, C. M., Clawson, L., Chaudhry, V., and Diette, G. B.	2001	Hospitalization in amyotrophic lateral sclerosis: Causes, costs, and outcomes	Neurology
Lee, J. E., Goo, A., Shin, D. W., and Yoo, J. H.	2019	Korean medical professionals' attitudes and experiences on advance care planning for noncancerous disease	Annals of Geriatric Medicine and Research
Lemoignan, J., and Ells, C.	2010	Amyotrophic lateral sclerosis and assisted ventilation: How patients decide	Palliative And Supportive Care
Lerum, S. V., Solbraekke, K. N., and Frich, J. C.	2016	Family caregivers' accounts of caring for a family member with motor neurone disease in Norway: a qualitative study	BMC Palliative Care
Lerum, S. V., Solbraekke, K. N., and Frich, J. C.	2017	Healthcare professionals' accounts of challenges in managing motor neurone disease in primary healthcare: a qualitative study	Health And Social Care In The Community
Lerum, S. V., Solbraekke, K. N., Holmøy, T., and Frich, J. C.	2015	Unstable terminality: negotiating the meaning of chronicity and terminality in motor neurone disease	Sociology Of Health And Illness

Levi, B. H., Simmons, Z., Hanna, C., Brothers, A., Lehman, E., Farace, E., Bain, M., Stewart, R., and Green, M. sJ.	2017	Advance care planning for patients with amyotrophic lateral sclerosis	Amyotrophic Lateral Sclerosis And Frontotemporal Degeneration
Linse, K., Rüger, W., Joos, M., Schmitz-Peiffer, H., Storch, A., and Hermann, A.	2018	Usability of eyetracking computer systems and impact on psychological wellbeing in patients with advanced amyotrophic lateral sclerosis	Amyotrophic Lateral Sclerosis And Frontotemporal Degeneration
Linse, K., Rüger, W., Joos, M., Schmitz-Peiffer, H., Storch, A., and Hermann, A.	2017	Eye-tracking-based assessment suggests preserved well-being in locked-in patients	Annals Of Neurology
Lulé, D., Häcker, S., Ludolph, A., Birbaumer, N., and Kübler, A.	2008	Depression and quality of life in patients with amyotrophic lateral sclerosis	Deutsches Arzteblatt International
Lulé, D., Nonnenmacher, S., Sorg, S., Heimrath, J., Mautzinger, M., Meyer, T., Kübler, A., Birbaumer, N., and Ludolph, A. C.	2014	Live and let die: Existential decision processes in a fatal disease	Journal Of Neurology
Lulé, D., Ehlich, B., Lang, D., Sorg, S., Heimrath, J., Kübler, A., Birbaumer, N., and Ludolph, A. C.	2013	Quality of life in fatal disease: the flawed judgement of the social environment	Journal Of Neurology
Maessen, M., Veldink, J. H., Onwuteaka-Philipsen, B. D., de Vries, J. M., Wokke, J. H. J., van der Wal, G., and van den Berg, L. H.	2009	Trends and determinants of end-of-life practices in ALS in the Netherlands	Neurology
Maessen, M., Veldink, J. H., Onwuteaka-Philipsen, B. D., Hendricks, H. T., Schelhaas, H. J., Grupstra, H. F., van der Wal, G., and van den Berg, L. H.	2014	Euthanasia and physician-assisted suicide in amyotrophic lateral sclerosis: a prospective study	Journal Of Neurology
Maetens, A., Deliens, L., De Bleecker, J., Caraceni, A., De Ridder, M., Beernaert, K., and Cohen, J.	2019	Healthcare utilization at the end of life in people dying from amyotrophic lateral sclerosis: A retrospective cohort study using linked administrative data	Journal of the Neurological Sciences

Mandler, R. N., Anderson Jr, F. A., Miller, R. G., Clawson, L., Cudkowicz, M., Del Bene, M., Bradley, W. G., Boynton de Sepulveda, L. I., Brooks, B. R., Cashman, N. R., Graves, M., Harati, Y., Heiman-Patterson, T., Lyon, M., Mitsumoto, H., Moore, D., Ringel, S. P., Rosenfeld, J., Ross, M. A., Strong, M. J., and Sufit, R. L.	2001	The ALS patient care database: Insights into end-of-life care in ALS	Amyotrophic Lateral Sclerosis And Other Motor Neuron Disorders
Mandrioli, J., Faglioni, P., Nichelli, P., and Sola, P.	2006	Amyotrophic lateral sclerosis: Prognostic indicators of survival	Amyotrophic Lateral Sclerosis
Marchese, S., Lo Coco, D., and Lo Coco, A.	2008	Outcome and attitudes toward home tracheostomy ventilation of consecutive patients: A 10-year experience	Respiratory Medicine
Marin, B., Beghi, E., Vial, C., Bernard, E., Lautrette, G., Clavelou, P., Guy, N., Lemasson, G., Debruxelles, S., Cintas, P., Antoine, J.C., Camdessanche, J.P., Logroscino, G., Preux, P.M., and Couratier, P.	2016	Evaluation of the application of the European guidelines for the diagnosis and clinical care of amyotrophic lateral sclerosis (ALS) patients in six French ALS centres	European Journal Of Neurology
Martin, J., and Turnbull, J.	2001	Lasting impact in families after death from ALS	Amyotrophic Lateral Sclerosis And Other Motor Neuron Disorders
Martin, N.H., Lawrence, V., Murray, J., Janssen, A., Higginson, I., Lyall, R., Burman, R., Leigh, P.N., Al-Chalabi, A., and Goldstein, L.H.	2016	Decision Making About Gastrostomy and Noninvasive Ventilation in Amyotrophic Lateral Sclerosis	Qualitative Health Research
Martin, N. H., Landau, S., Janssen, A., Lyall, R., Higginson, I., Burman, R., McCrone, P., Sakel, M., Ellis, C. M., Shaw, C. E., Al-Chalabi, A., Leigh, P. N., and Goldstein, L. H.	2014	Psychological as well as illness factors influence acceptance of non-invasive ventilation (NIV) and gastrostomy in amyotrophic lateral sclerosis (ALS): A prospective population study	Amyotrophic Lateral Sclerosis And Frontotemporal Degeneration
Mazzini, L., Corra, T., Zaccala, M., Mora, G., Del Piano, M., and Galante, M.	1995	Percutaneous endoscopic gastrostomy and enteral nutrition in amyotrophic lateral sclerosis	Journal Of Neurology

Mc Veigh, C., Donaghy, C., Mc Laughlin, B., Dick, A., Kaur, K., Mc Conville, J., and Watson, M.	2019	Palliative care for patients with motor neurone disease and their bereaved carers: a qualitative study	BMC Palliative Care
McDermott, C. J., Shaw, P. J., Stavroulakis, T., Walters, S. J., Al-Chalabi, A., Chandran, S., Crawley, F., Dick, D., Donaghy, C., Eames, P., Fish, M., Gent, C., Gorrie, G., Hamdalla, H., Hanemann, C. O., Johnson, M., Majeed, T., Malaspina, A., Morrison, K., Orrell, R., Pinto, A., Radunovic, A., Roberts, M., Talbot, K., Turner, M. R., Williams, T., Young, C., and ProGas Study Group	2015	Gastrostomy in patients with amyotrophic lateral sclerosis (ProGas): a prospective cohort study	Lancet Neurology
McDonald, E. R., Walter, R. A., Wiedenfeld, S. A., Hillel, A., and Carpenter, C. L.	1994	Survival in amyotrophic lateral sclerosis: The role of psychological factors	Archives Of Neurology
McKelvey, M., Beukelman, D., Kawai, N., and Evans, D.	2013	Nine Suggestions for the Professional Support of Persons With Amyotrophic Lateral Sclerosis as Recounted by Surviving Spouses: A Phenomenological Study	Perspectives On Augmentative And Alternative Communication
McKelvey, M., Evans, D. L., Kawai, N., and Beukelman, D.	2012	Communication Styles of Persons with ALS as Recounted by Surviving Partners	Augmentative And Alternative Communication
McKim, D. A., King, J., Walker, K., Leblanc, C., Timpson, D., Wilson, K. G., Marks, M., Curran, D., and Woolnough, A.	2012	Formal ventilation patient education for ALS predicts real-life choices	Amyotrophic Lateral Sclerosis
Messori, A., Trippoli, S., Becagli, P., and Zaccara, G.	1999	Cost effectiveness of riluzole in amyotrophic lateral sclerosis	Pharmacoeconomics
Miller, R. G., Anderson, F. A., Jr., Bradley, W. G., Brooks, B. R., Mitsumoto, H., Munsat, T. L., and Ringel, S. P.	2000	The ALS patient care database: goals, design, and early results. ALS C.A.R.E. Study Group	Neurology

Moss, A. H., Oppenheimer, E. A., Casey, P., Cazzolli, P. A., Roos, R. P., Stocking, C. B., and Siegler, M.	1996	Patients with amyotrophic lateral sclerosis receiving long-term mechanical ventilation - Advance care planning and outcomes	Chest
Munan, M., Luth, W., Genuis, S.K., Johnston, W.S., and MacIntyre, E.	2020	Transitions in Amyotrophic Lateral Sclerosis: Patient and Caregiver Experiences	The Canadian Journal of Neurological Science
Murphy, P. L., Albert, S. M., Weber, C. M., Del Bene, M. L., and Rowland, L. P.	2000	Impact of spirituality and religiousness on outcomes in patients with ALS	Neurology
Mustfa, N., Walsh, E., Bryant, V., Lyall, R. A., Addington-Hall, J., Goldstein, L. H., Donaldson, N., Polkey, M. I., Moxham, J., and Leigh, P. N.	2006	The effect of noninvasive ventilation on ALS patients and their caregivers	Neurology
Neudert, C., Oliver, D., Wasner, M., and Borasio, G. D.	2001	The course of the terminal phase in patients with amyotrophic lateral sclerosis	Journal Of Neurology
Neudert, C., Wasner, M., and Borasio, G. D.	2001	Patients' assessment of quality of life instruments: A randomised study of SIP, SF-36 and SEIQoL-DW in patients with amyotrophic lateral sclerosis	Journal Of The Neurological Sciences
Ng, L., Talman, P., and Khan, F.	2011	Motor neurone disease: disability profile and service needs in an Australian cohort	International Journal Of Rehabilitation Research
Nolan, M. T., Hughes, M. T., Kub, J., Terry, P. B., Astrow, A., Thompson, R. E., Clawson, L., Texeira, K., and Sulmasy, D. P.	2009	Development and validation of the Family Decision-Making Self-Efficacy Scale	Palliative And Supportive Care
Nolan, M. T., Kub, J., Hughes, M. T., Terry, P. B., Astrow, A. B., Carbo, C. A., Thompson, R. E., Clawson, L., Texeira, K., and Sulmasy, D. P.	2008	Family health care decision making and self-efficacy with patients with ALS at the end of life	Palliative And Supportive Care
Ohnsorge, K., Rehmann-Sutter, C., Streeck, N., and Gudat, H.	2019	Wishes to die at the end of life and subjective experience of four different typical dying trajectories. A qualitative interview study	PloS One
Oliver, D.	1996	The quality of care and symptom control - the effects on the terminal phase of ALSMND	Journal Of The Neurological Sciences

Olney, R. K., Murphy, J., Forshew, D., Garwood, E., Miller, B. L., Langmore, S., Kohn, M. A., and Lomen-Hoerth, C.	2005	The effects of executive and behavioral dysfunction on the course of ALS	Neurology
Ozanne, A. O., Graneheim, U. H., and Strang, S.	2013	Finding meaning despite anxiety over life and death in amyotrophic lateral sclerosis patients	Journal Of Clinical Nursing
Peretti-Watel, P., Bendiane, M.-K., Galinier, A., Favre, R., Ribiere, C., Lapiana, J-M., and Obadia, Y.	2008	District nurses' attitudes toward patient consent: The case of mechanical ventilation on amyotrophic lateral sclerosis patients: Results from a French national survey	Journal Of Critical Care
Preston, H., Fineberg, I. C., Callagher, P., and Mitchell, D. J.	2012	The Preferred Priorities for Care document in Motor Neurone Disease: Views of bereaved relatives and carers	Palliative Medicine
Rabkin, J. G., Albert, S. M., Del Bene, M. L., O'Sullivan, I., Tider, T., Rowland, L. P., and Mitsumoto, H.	2005	Prevalence of depressive disorders and change over time in late-stage ALS	Neurology
Rabkin, J. G., Albert, S. M., Rowland, L. P., and Mitsumoto, H.	2009	How common is depression among ALS caregivers? A longitudinal study	Amyotrophic Lateral Sclerosis
Rabkin, J. G., Albert, S. M., Tider, T., Del Bene, M. L., O'Sullivan, I., Rowland, L. P., and Mitsumoto, H.	2006	Predictors and course of elective long-term mechanical ventilation: A prospective study of ALS patients	Amyotrophic Lateral Sclerosis
Rabkin, J. G., Goetz, R., Factor-Litvak, P., Hupf, J., McElhiney, M., Singleton, J., and Mitsumoto, H.	2015	Depression and wish to die in a multicenter cohort of ALS patients	Amyotrophic Lateral Sclerosis And Frontotemporal Degeneration
Rabkin, J. G., Wagner, G. J., and Del Bene, M.	2000	Resilience and distress among amyotrophic lateral sclerosis patients and caregivers	Psychosomatic Medicine
Rabkin, J., Ogino, M., Goetz, R., McElhiney, M., Hupf, J., Heitzman, D., Heiman-Patterson, T., Miller, R., Katz, J., Lomen-Hoerth, C., Imai, T., Atsuta, N., Morita, M., Tateishi, T., Matsumura, T., and Mitsumoto, H.	2014	Japanese and American ALS patient preferences regarding TIV (tracheostomy with invasive ventilation): a cross-national survey	Amyotrophic Lateral Sclerosis And Frontotemporal Degeneration

Rabkin, J., Ogino, M., Goetz, R., McElhiney, M., Marziliano, A., Imai, T., Atsuta, N., Morita, M., Tateishi, T., Matsumura, T., and Mitsumoto, H.	2013	Tracheostomy with invasive ventilation for ALS patients: Neurologists' roles in the US and Japan	Amyotrophic Lateral Sclerosis And Frontotemporal Degeneration
Rabkin, J., Goetz, R., Murphy, J. M. Factor-Litvak, P., Mitsumoto, H., and ALS COSMOS Study Group	2016	Cognitive impairment, behavioral impairment, depression, and wish to die in an ALS cohort	Neurology
Ray, R. A., and Street, A. F.	2006	Caregiver bodywork: family members' experiences of caring for a person with motor neurone disease	Journal Of Advanced Nursing
Robbins, R. A., Simmons, Z., Bremer, B. A., Walsh, S. M., and Fischer, S.	2001	Quality of life in ALS is maintained as physical function declines	Neurology
Roscoe, L. A., Malphurs, J. E., Dragovic, L. J., and Cohen, D.	2003	Antecedents of euthanasia and suicide among older women	Journal Of The American Medical Women's Association
Rosengren, K., Gustafsson, I., and Jarnevi, E.	2015	Every Second Counts: Women’s Experience of Living With ALS in the End-of-Life Situations	Home Health Care Management And Practice
Ruffell, T. O., Martin, N. H., Janssen, A., Wijesekera, L., Knights, C., Burman, R., Oliver, D. J., Al-Chalabi, A., and Goldstein, L. H.	2013	Healthcare professionals' views on the provision of gastrostomy and noninvasive ventilation to amyotrophic lateral sclerosis patients in England, Wales, and Northern Ireland	Journal Of Palliative Care
Russell, J. A., Williams, M. A., and Drogan, O.	2010	Sedation for the imminently dying: survey results from the AAN Ethics Section	Neurology
Sancho, J., Servera, E., Diaz, J. L., Banuls, P., and Marin, J.	2011	Home tracheotomy mechanical ventilation in patients with amyotrophic lateral sclerosis: Causes, complications and 1-year survival	Thorax
Sato, K., Morimoto, N., Deguchi, K., Ikeda, Y., Matsuura, T., and Abe, K.	2014	Seven amyotrophic lateral sclerosis patients diagnosed only after development of respiratory failure	Journal Of Clinical Neuroscience
Schellenberg, K. L., Schofield, S. J., Fang, S., and Johnston, W. S.	2014	Breaking bad news in amyotrophic lateral sclerosis: The need for medical education	Amyotrophic Lateral Sclerosis And Frontotemporal Degeneration

Seeber, A. A., Pols, A. J., Hijdra, A., Grupstra, H. F., Willems, D. L., and de Visser, M.	2019	Advance care planning in progressive neurological diseases: lessons from ALS	BMC Palliative Care
Segerstrom, S. C., Kasarskis, E. J., Fardo, D. W., and Westgate, P. M.	2019	Socioemotional selectivity and psychological health in amyotrophic lateral sclerosis patients and caregivers: a longitudinal, dyadic analysis	Psychology & Health
Sharma, R. K., Hughes, M. T., Nolan, M. T., Tudor, C., Kub, J., Terry, P. B., and Sulmasy, D. P.	2011	Family understanding of seriously-ill patient preferences for family involvement in healthcare decision making	Journal Of General Internal Medicine
Silverstein, M. D., Stocking, C. B., Antel, J. P., Beckwith, J., Roos, R. P., and Siegler, M.	1991	Amyotrophic lateral sclerosis and life-sustaining therapy: patients' desires for information, participation in decision making, and life-sustaining therapy	Mayo Clinic Proceedings
Simmons, Z., Bremer, B. A., Robbins, R. A., Walsh, S. M., and Fischer, S.	2000	Quality of life in ALS depends on factors other than strength and physical function	Neurology
Solomon, D. N., and Hansen, L.	2015	Living through the end: The phenomenon of dying at home	Palliative And Supportive Care
Spataro, R., and La Bella, V.	2021	The capacity to consent to treatment in amyotrophic lateral sclerosis: a preliminary report	Journal of Neurology
Stavroulakis, T., Baird, W. O., Baxter, S. K., Walsh, T., Shaw, P. J., and McDermott, C. J.	2014	Factors influencing decision-making in relation to timing of gastrostomy insertion in patients with motor neurone disease	BMJ Supportive And Palliative Care
Steinhauser, K. E., Olsen, A., Johnson, K. S., Sanders, L. L., Olsen, M., Ammarell, N., and Grossoehme, D.	2016	The feasibility and acceptability of a chaplain-led intervention for caregivers of seriously ill patients: A Caregiver Outlook pilot study	Palliative And Supportive Care
Stutzki, R., Schneider, U., Reiter-Their, S., and Weber, M.	2012	Attitudes toward assisted suicide and life-prolonging measures in Swiss ALS patients and their caregivers	Frontiers In Psychology
Stutzki, R., Weber, M., Reiter-Theil, S., Simmen, U., Borasio, G. D., and Jox, R. J.	2014	Attitudes towards hastened death in ALS: a prospective study of patients and family caregivers	Amyotrophic Lateral Sclerosis And Frontotemporal Degeneration

Sulmasy, D. P., Terry, P. B., Weisman, C. S., Miller, D. J., Stallings, R. Y., Vettese, M. A., and Haller, K. B.	1998	The accuracy of substituted judgments in patients with terminal diagnoses	Annals Of Internal Medicine
Sulmasy, D.P., Hughes, M.T., Yenokyan, G., Kub, J., Terry, P.B., Astrow, A.B., Johnson, J.A., Ho, G., and Nolan, M.T.	2017	The Trial of Ascertaining Individual Preferences for Loved Ones' Role in End-of-Life Decisions (TAILORED) Study: A Randomized Controlled Trial to Improve Surrogate Decision Making	Journal Of Pain And Symptom Management
Sulmasy, D. P., Hughes, M. T., Thompson, R. E., Astrow, A. B., Terry, P. B., Kub, J., and Nolan, M. T.	2007	How would terminally ill patients have others make decisions for them in the event of decisional incapacity? A longitudinal study	Journal Of The American Geriatrics Society
Kim, S., Chung, S.E., Lee, S., Park, J., Choi, S., and Kim, S.	2016	Experience of complementary and alternative medicine in patients with amyotrophic lateral sclerosis and their families: A qualitative study	Amyotrophic Lateral Sclerosis And Frontotemporal Degeneration
Tagami, M., Kimura, F., Nakajima, H., Ishida, S., Fujiwara, S., Doi, Y., Hosokawa, T., Yamane, K., Unoda, K., Hirose, T., Tani, H., Ota, S., Ito, T., Sugino, M., Shinoda, K., and Hanafusa, T.	2014	Tracheostomy and invasive ventilation in Japanese ALS patients: Decision-making and survival analysis: 1990-2010	Journal Of The Neurological Sciences
Tandan, R., Boylan, K., Levine, T., Lomen-Hoerth, C., Callas, P., Maginnis, K., and Lyon, M.	2014	Prospective study of cost of care at multidisciplinary centers adhering to aan amyotrophic lateral sclerosis (ALS) practice parameters	Neurology
Thurn, T., Borasio, G. D., Chiò, A., Galvin, M., McDermott, C. J., Mora, G., Sermeus, W., Winkler, A. S., and Anneser, J.	2019	Physicians' attitudes toward end-of-life decisions in amyotrophic lateral sclerosis	Amyotrophic Lateral Sclerosis And Frontotemporal Degeneration
Tobin, K., Maguire, S., Corr, B., Normand, C., Hardiman, O., and Galvin, M.	2021	Discrete choice experiment for eliciting preference for health services for patients with ALS and their informal caregivers	BMC Health Services Research

Trail, M., Nelson, N. D., Van, J. N., Appel, S. H., and Lai, E. C.	2003	A study comparing patients with amyotrophic lateral sclerosis and their caregivers on measures of quality of life, depression, and their attitudes toward treatment options	Journal Of The Neurological Sciences
Ushikubo, M.	2015	Comparison Between Home and Hospital as the Place of Death for Individuals With Amyotrophic Lateral Sclerosis in the Last Stages of Illness	American Journal Of Hospice And Palliative Medicine
Ushikubo, M., and Okamoto, K.	2012	Circumstances surrounding death and nursing difficulties with end-of-life care for individuals with ALS in central Japan	International Journal Of Palliative Nursing
van Eenennaam, R. M., Kruithof, W. J., van Es, M. A., Kruitwagen-van Reenen, E. T., Westeneng, H. -J., Visser-Meily, J. M. A., van den Berg, L. H., and Beelen, A.	2020	Discussing personalized prognosis in amyotrophic lateral sclerosis: development of a communication guide	BMC Neurology
Veldink, J. H., Wokke, J. H. J., Van Der Wal, G., De Vianney Jong, J. M. B., and Van Den Berg, L. H.	2002	Euthanasia and physician-assisted suicide among patients with amyotrophic lateral sclerosis in the Netherlands	New England Journal Of Medicine
Vianello, A., Arcaro, G., Palmieri, A., Ermani, M., Braccioni, F., Gallan, F., Soraru, G., and Pegoraro, E.	2011	Survival and quality of life after tracheostomy for acute respiratory failure in patients with amyotrophic lateral sclerosis	Journal Of Critical Care
Vignola, A., Guzzo, A., Calvo, A., Moglia, C., Pessia, A., Cavallo, E., Cammarosano, S., Giacone, S., Ghiglione, P., and Chio, A.	2008	Anxiety undermines quality of life in ALS patients and caregivers	European Journal Of Neurology
Vitacca, M., and Vianello, A.	2013	Respiratory outcomes of patients with amyotrophic lateral sclerosis: An italian nationwide survey	Respiratory Care
Vitale, A., and Genge, A.	2007	Codman Award 2006: the experience of hope in ALS patients	Axone
Wales, J., Isenberg, S. R., Wegier, P., Shapiro, J., Cellarius, V., Buchman, S., Husain, A., and Khoshnood, N.	2018	Providing Medical Assistance in Dying within a Home Palliative Care Program in Toronto, Canada: An Observational Study of the First Year of Experience	Journal of Palliative Medicine

Walter, H. A. W., Seeber, A. A., Willems, D. L., and de Visser, M.	2018	The Role of Palliative Care in Chronic Progressive Neurological Diseases—A Survey Amongst Neurologists in the Netherlands	Frontiers in Neurology
Wang, L. H., Elliott, M. A., Jung Henson, L., Gerena-Maldonado, E., Strom, S., Downing, S., Vetrovs, J., Kayihan, P., Paul, P., Kennedy, K., Benditt, J. O., and Weiss, M. D	2016	Death with dignity in Washington patients with amyotrophic lateral sclerosis	Neurology
Warner, T. D., Roberts, L. W., Smithpeter, M., Rogers, M., Roberts, B., McCarty, T., Franchini, G., Geppert, C., and Obenshain, S. S	2001	Uncertainty and opposition of medical students toward assisted death practices	Journal Of Pain And Symptom Management
Wasner, M., Klier, H., and Borasio, G. D.	2001	The use of alternative medicine by patients with amyotrophic lateral sclerosis	Journal Of The Neurological Sciences
Weber, C., Fijalkowska, B., Ciecwierska, K., Lindblad, A., Badura-Lotter, G., Andersen, P.M., Kuźma-Kozakiewicz, M., Ludolph, A.C., Lulé, D., Pasierski, T., and Lynöe, N.	2017	Existential decision-making in a fatal progressive disease: how much do legal and medical frameworks matter?	BMC Palliative Care
Westaby, J. D., Versenyi, A., and Hausmann, R. C.	2005	Intentions to work during terminal illness: An Exploratory study of antecedent conditions	Journal Of Applied Psychology
Wiebe, E., Shaw, J., Green, S., Trouton, K., and Kelly, M.	2018	Reasons for requesting medical assistance in dying	Canadian Family Physician
Winther, D., Lorenzen, C.K., and Dreyer, P.	2020	Everyday life experiences of close relatives of people with amyotrophic lateral sclerosis receiving home mechanical ventilation—A qualitative study	Journal of Clinical Nursing
Young, J. M., Marshall, C. L., and Anderson, E. J.	1994	Amyotrophic lateral sclerosis patients' perspectives on use of mechanical ventilation	Health And Social Work
Zhang, L., Sanders, L., and Fraser, R. J. L.	2012	Nutritional support teams increase percutaneous endoscopic gastrostomy uptake in motor neuron disease	World Journal Of Gastroenterology

Zwicker, J., Qureshi, D., Talarico, R., Bourque, P., Scott, M., Chin-Yee, N., and Tanuseputro, P.	2019	Dying of amyotrophic lateral sclerosis: Health care use and cost in the last year of life	Neurology
